# Nanoscale imaging of the adhesion core including integrin β1 on intact living cells using scanning electron-assisted dielectric-impedance microscopy

**DOI:** 10.1371/journal.pone.0204133

**Published:** 2018-09-20

**Authors:** Tomoko Okada, Toshihiko Ogura

**Affiliations:** Biomedical Research Institute, National Institute of Advanced Industrial Science and Technology (AIST), Higashi, Tsukuba, Ibaraki, Japan; Leiden University, NETHERLANDS

## Abstract

The integrins are a superfamily of transmembrane proteins composed of α and β subunit dimers involved in cell–cell and cell–extracellular matrix interactions. The largest integrin subgroup is integrin β1, which contributes to several malignant phenotypes. Recently, we have developed a novel imaging technology named scanning electron-assisted dielectric-impedance microscopy (SE-ADM), which visualizes untreated living mammalian cells in aqueous conditions with high contrast. Using the SE-ADM system, we observed 60-nm gold colloids with antibodies directly binding to the focal adhesion core containing integrin β1 on mammalian cancer cells without staining and fixation. The adhesion core contains three or four high-density regions of integrin β1 and connects to the actin filament. An adhesion core with high-density integrin β1 is suggested to contain 10–20 integrin dimers. Our SE-ADM system can also visualize various other membrane proteins in living cells in medium without staining and fixation.

## Introduction

Cell adhesion is mediated by various transmembrane adhesion molecules. Most cell–cell and cell–extracellular matrix interactions involve integrins, a superfamily of transmembrane heterodimeric proteins consisting of α and β subunits [1–4]. The largest integrin subgroup is integrin β1, which contributes to several malignant phenotypes (proliferation, migration, metastasis/invasion, and angiogenesis) in a wide variety of cancers, such as breast, colon, and ovarian cancers [5–7]. Moreover, integrin β1 is a candidate therapeutic target in many solid cancers, and its overexpression in colorectal tumors is associated with poor prognosis [8, 9].

The 2D and 3D structures of integrin-containing focal adhesion cores have been observed by super-resolution fluorescence optical microscopy [10, 11], atomic force microscopy (AFM) [12, 13], and electron microscopy (EM) [14–17]. According to single-particle analysis of EM images, the conformational structure of purified integrin α5β1 is approximately 20 nm long and 15 nm wide [17]. Furthermore, super-resolution optical microscopy has revealed the 3D structures of the focal adhesion domain in fluorescently labeled cells [10, 11]. However, as the cells in these reports were fluorescently labeled and/or fixed, the structures of the adhesion-core domain in intact living cells remain unknown.

Mammalian cells in medium have been observed by atmospheric scanning electron microscopy (SEM) [18–20], but conventional SEM systems cause radiation damage to the specimens [21]. Recently, we have developed a novel imaging technology named scanning electron-assisted dielectric-impedance microscopy (SE-ADM), which visualizes various intact biological specimens in aqueous conditions with minimal radiation-induced damage (S1 Fig) [22–24]. The biological samples are enclosed in a liquid holder composed of tungsten (W)-coated silicon nitride (SiN) film, which shields them from the electron beam (EB). Most of the irradiated electrons are absorbed by the W layer on the upper SiN thin film, increasing the negative electric field potential in this locality [22]. The negative potential is detected through the specimen in water at the bottom measurement terminal. As water has a high electric permittivity, the electric potential induced by the irradiated electrons in the W-coated film is propagated to the lower SiN film through the sample solution [22]. On the contrary, as biological specimens contain organic materials with low electric permittivity, they decrease the transmitted electric signal [22]. Therefore, our system can facilitate high-contrast imaging with low radiation-induced damage.

In the first application of our SE-ADM system, we observed untreated living mammalian cells in aqueous conditions [25]. Next, we clearly observed antibody-binding nanobeads in the liquid phase [26]. We also directly detected streptavidin-conjugated nanobeads binding to untreated cells in medium via a biotin-conjugated anti-CD44 antibody [26]. Moreover, our system obtains clear and simultaneous images of cellular organelles and nanobeads in cells without metal and/or fluorescence staining [26]. The present study reports the first SE-ADM observations of cell adhesion core domains containing integrin β1 molecules on intact, unfixed living cells. We also observe 60-nm gold colloids directly binding to integrin β1 via antibodies targeting the focal adhesion cores in mammalian cells.

## Results

**Fig 1:**
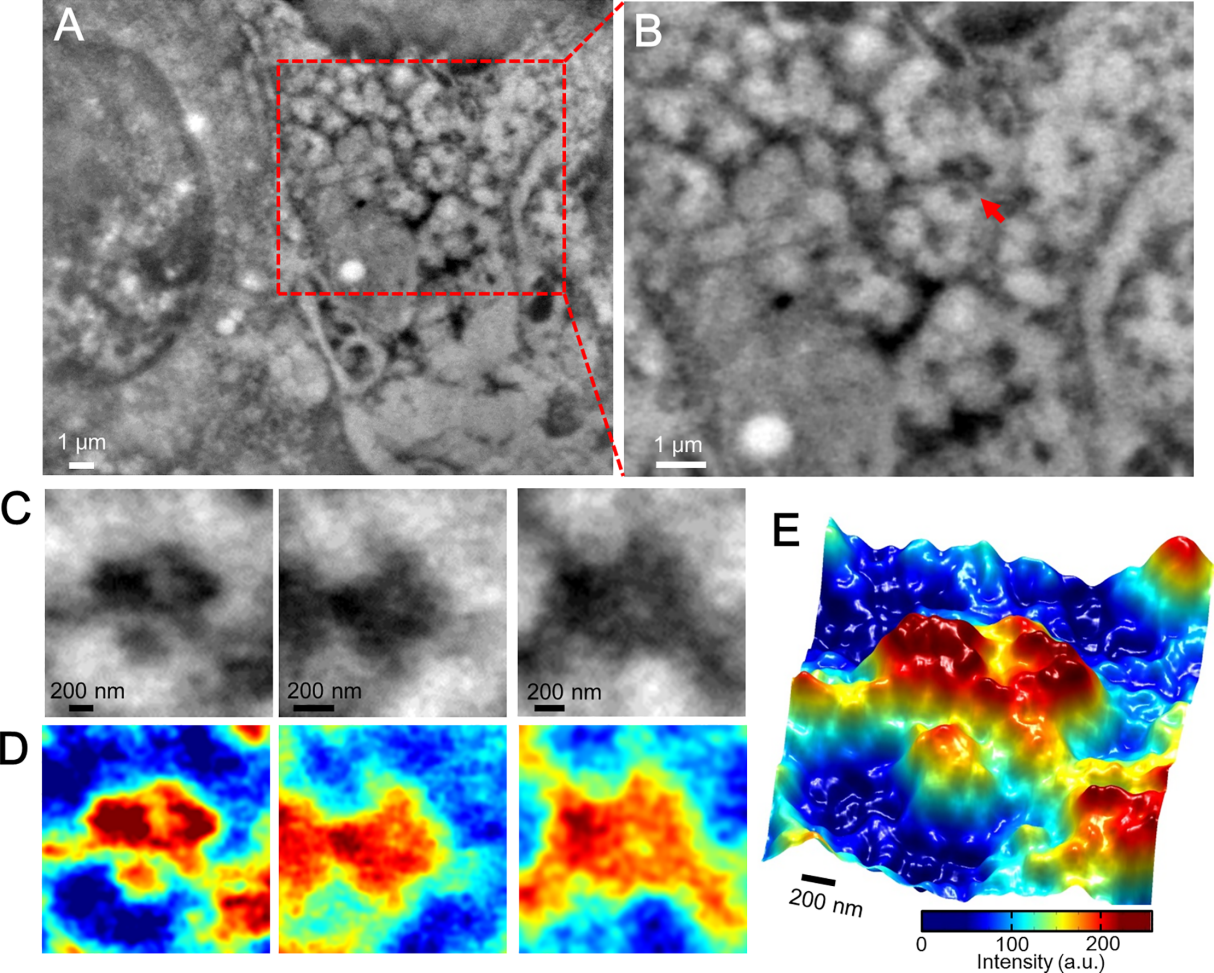
Scanning electron-assisted dielectric microscopy images of the cell adhesion core of mammalian cancer cells in the medium. (A) Dielectric impedance image of untreated 4T1E/M3 cells in medium (5,000× magnification, 7-kV electron beam, −9 V bias). The large sphere on the left is the nucleus, and mesh-like structures with dense black granule clusters are shown on the right. (B) Enlarged image of the red boxed area in (A) showing the high-density black granule clusters linked to the mesh-like structures. (C) Three enlarged images of the black granule clusters denoted by the red arrow in (B) and other imaging areas. (D) Pseudo-color maps of (C) after intensity inversion. (E) 3D color map of the left side of (D).

Mouse breast cancer 4T1E/M3 cells [27–29] were cultured in a culture dish containing a medium-loaded 50-nm SiN holder [25]. After 4–5 days of culture, the cells formed a confluent monolayer on the SiN film of the holder. The holder containing the cells was separated from the plastic culture dish and attached to an acrylic holder, which was installed in the SE-ADM system (S1 Fig) [24]. The cultured cancer cells in the space between the SiN films were kept under atmospheric pressure. An image of mammalian cells taken by the SE-ADM system (5,000× magnification) clearly shows the intracellular structure, nucleus, and spherical vesicles (Fig 1A). The image clarifies the complex membrane structure with many dispersed characteristic high-contrast high-density black granules on the right side of the nucleus (Fig 1A). An enlarged image of the red box in Fig 1A reveals clear black granules and a mesh-like structure (Fig 1B). The 4T1E/M3 cells chosen for this study strongly express integrin β1, as reported in our earlier study [27]. The characteristic high-contrast dense black granules in the images were assumed as integrin-containing focal adhesion cores [1, 2, 10]. Because integrin adhesion cores are reportedly connected to actin filaments [1, 2, 10], we assumed that the mesh-like structure comprised actin filaments. Fig 1C is a magnified image of three high-density black granules connected to the filaments indicated by the red arrow in Fig 1B and other scanned areas. For a detailed analysis, Fig 1D displays the pseudo-color maps after intensity inversion. The magnified images (Fig 1C and 1D) and 3D map (Fig 1E) reveal that the high-density granules consist of three or four spherical particles connected to the filament structures. Because the cell adhesion structure is known to comprise a high-density region of many proteins, including integrins and actin filaments [1, 2, 10], we speculated that the imaged high-density regions are cellular adhesion structures. To analyze the integrin expression in 4T1E/M3 cancer cells, we stained the cells with rabbit anti-integrin β1 antibody and FITC-conjugated anti-rabbit IgG and observed them by optical fluorescent microscopy. Phase contrast (S2A Fig) and fluorescence (S2B Fig) images clearly reveal that integrin β1 proteins were widely expressed on the cellular membranes. An enlarged image of the nuclear peripheral region (red boxed area in S2B Fig) exhibits many spherical fluorescent spots of various sizes (S2C Fig).

**Fig 2:**
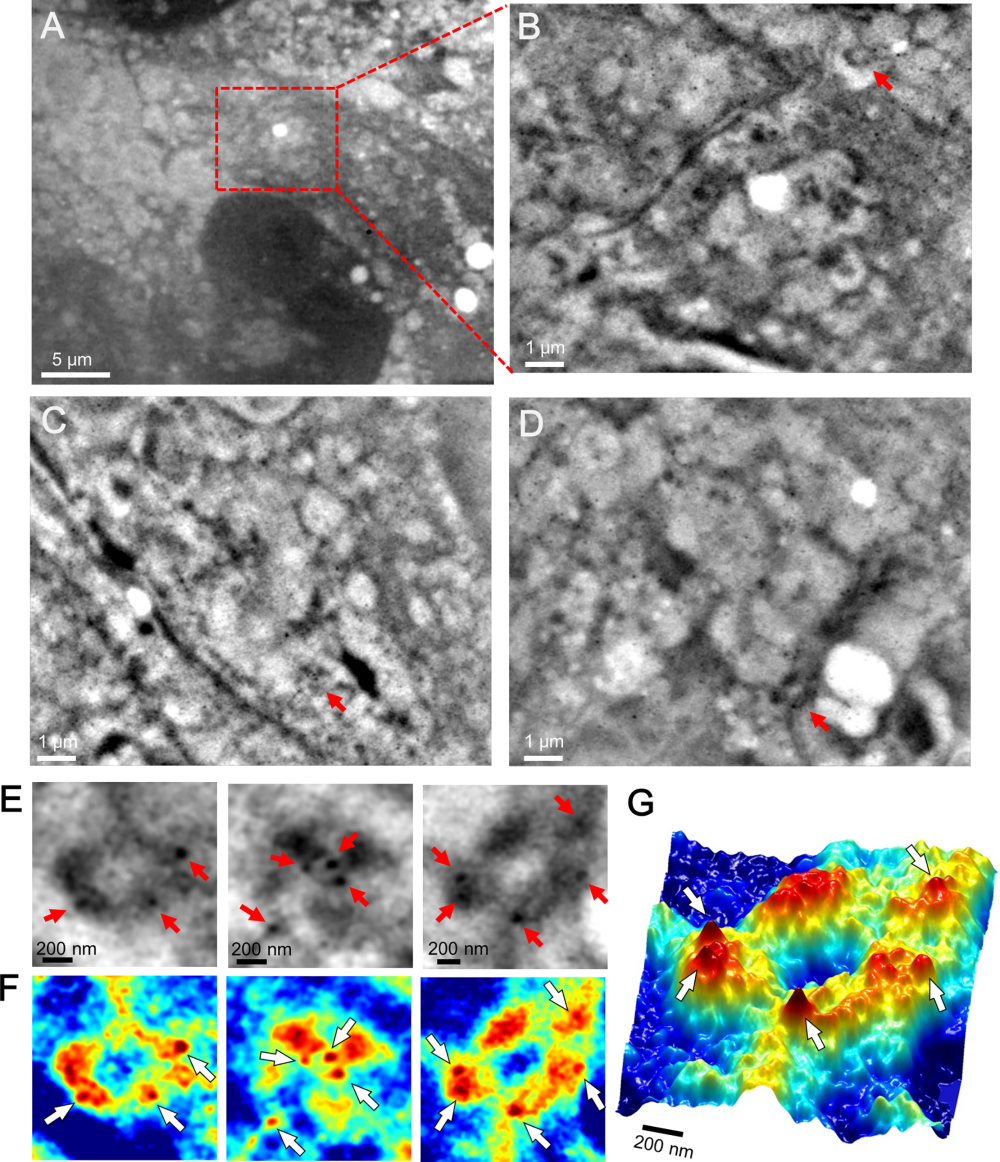
Scanning electron-assisted dielectric microscopy images of the cell adhesion cores of mammalian cancer cells with 60-nm gold colloids in the medium. (A) Dielectric impedance image of 4T1E/M3 cells with 60-nm gold colloid bound to integrin cores in the medium (1,500× magnification, 7 kV electron beam, −9 V bias). The large sphere at the bottom is the nucleus. (B) High-magnification (10,000×, 7 kV) image of the red-boxed area in (A) showing the high-density black granule clusters with 60-nm gold colloids linked to the mesh-like structures. (C–D) High-magnification (10,000×, 7 kV) images at other cell positions. (E) Three enlarged images of the black granule clusters with 60-nm gold colloids bound to integrin β1 indicated by red arrows in (B–D). The red arrows in (E) indicate the 60-nm gold colloids. (F) Pseudo-color maps of (E) after intensity inversion. White arrows indicate 60-nm gold colloids. (G) 3D color map of the right image of (F). The 60-nm gold colloids (indicated by white arrows) are clearly visible. Scale bars: 5 μm in (A), 1 μm in (B–D), and 200 nm in (E–G).

Next, we sought to identify integrins on living cells using our SE-ADM system. The mouse cancer cells (4T1E/M3) were incubated with biotin-conjugated rabbit anti-integrin β1 antibody and then stained with streptavidin-conjugated 60-nm gold colloids. This treatment bound the 60-nm gold colloids to the adhesion cores containing integrin β1 proteins on the cell membranes, and they were detected by our SE-ADM system (Fig 2A–2D). We selected the high-contrast characteristic granules in the images localized on or near the mesh-like structures with clearly contrasted gold colloids. Fig 2A is a low-magnification SE-ADM image (1,500×) of the cancer cells stained by anti-integrin antibody and 60-nm gold colloids. For a detailed observation, the peripheral region of the cell (red boxed area in Fig 2A) was scanned at 10,000× magnification (Fig 2B). Other cell regions imaged at the same magnification are shown in Fig 2C and 2D. These images show mesh-like structures and dense black cores bounded by small black dots, which indicate the streptavidin-conjugated 60-nm gold colloids. SE-ADM images of streptavidin-conjugated 60-nm gold colloid alone are shown in S3A and S3B Fig. The 60-nm gold colloids were coated with streptavidin and showed clear black contrast in the SE-ADM images, suggesting that the conductive gold material exerted an electrostatic shielding effect with streptavidin covered insulation. Fig 2E and 2F show the enlarged images and color maps after intensity inversion around the binding sites (indicated by red arrows in Fig 2B–2D). The high-density structures containing three or four spherical granule clusters were linked to the filament-like structures (Fig 2B–2F). Furthermore, they were clearly co-localized with the 60-nm gold colloids binding to integrin β1 (Fig 2E and 2F, red and white arrows). Therefore, these high-density structures with filaments were identified as the focal adhesion structures containing integrin β1. The focal adhesion structures appeared as triangular or square-like clusters connected to the mesh-like filament structures (Figs 1B–1D and 2B–2F). This connection between the adhesion core and mesh filaments was highlighted in the 3D color map, and its structure was bound with 60-nm gold colloids (white arrows; Fig 2G). To confirm that the 60-nm gold colloids specifically bound to the adhesion cores, we observed control specimens of 4T1E/M3 cells stained by streptavidin-conjugated 60-nm gold colloids alone, without anti-integrin antibody (S4A–S4E Fig). Clearly, the gold colloids were unattached to the adhesion cores, confirming that they bound specifically to the integrin cores via anti-integrin β1 antibody in Fig 2.

**Fig 3:**
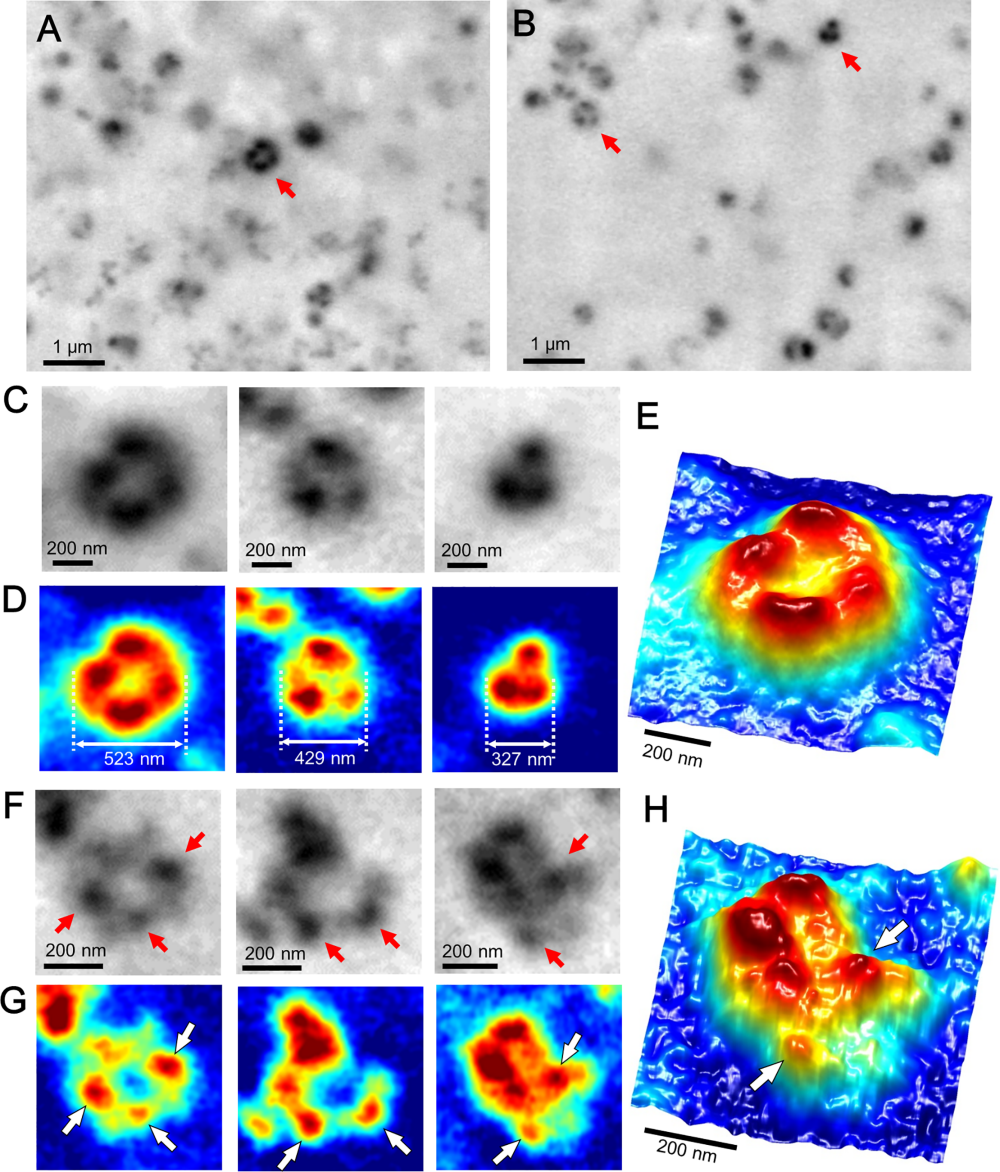
SE-ADM images of the focal adhesion core after cell detachment. (A) Dielectric impedance image of the cell adhesion core after removing the cells in the medium (10,000× magnification, 7-kV electron beam, −9 V bias). Clear black granule clusters are dispersed throughout the area. (B) Another dielectric impedance image of the cell adhesion core under the conditions of (A). (C) Three enlarged images of the black granule clusters indicated by red arrows in (A) and (B). (D) Pseudo-color maps of (C) after intensity inversion. (E) 3D color map of the left image of (D). The adhesion core contains clear clusters, including a few high-contrast granules. (F) Three enlarged images of the black granule clusters with 60-nm gold colloids and antibodies bound to integrin β1 after cell removal. Red arrows indicate the 60-nm gold colloids bound to integrin β1. (G) Pseudo-color maps of (F) after intensity inversion. White arrows indicate 60-nm gold colloids at the same positions as (F). (H) 3D color map of the right image of (G). White arrows indicate 60-nm gold colloids. Scale bars: 1 μm in (A–B) and 200 nm in (C–H).

We believe that the dense triangular- or square-shaped clusters (Figs 1E and 2G) were integrin-based adhesion cores and that the mesh-like filament structures were actin filaments. We speculated that removing the mesh-like structure would clarify the integrin-based adhesion core. We thus removed the cells and imaged the focal adhesion structure of integrin on the SiN film (Fig 3). The 4T1E/M3 confluent cell monolayers were softly detached from the film by positioning the acrylic holder and then opening the dish holder (S5A–S5C Fig). After removing the cells (S5D Fig), the Al holder was attached to a new acrylic holder (S5E and S5F Fig), and the liquid–holder set was installed in the SE-ADM system. Clear-contrast black granules of various sizes were observed across the scanned area (Fig 3A and 3B). For a detailed analysis, the dense black particles indicated by the red arrows in Fig 3A and 3B were enlarged and intensity-inverted into pseudo-color maps (Fig 3C and 3D). As shown in the 3D pseudo-color map (Fig 3E), the high-density structure consisted of three or four smooth spherical granules, which probably constitute the extracellular parts of the focal adhesion core. The mesh-like filament structures, which are suggested as the intracellular structures, are absent. The mean diameter of the spherical clusters (430 ± 56.1 nm) was calculated from nine selected adhesion clusters in Fig 3D and S6 Fig. When the cells were stained with anti-integrin β1 and FITC-conjugated secondary antibodies prior to detachment, fluorescent spots appeared in the images (S2D–S2F Fig). The sizes and shapes of the fluorescent spots after removing the cells (S2F Fig) resembled those of the spherical particle clusters in Fig 3A and 3B. This result suggests that, after cell detachment, integrin β1 and its connected proteins remained on the SiN-film surface in the dish holder or on the glass dish.

We also stained the cells with anti-integrin β1 antibody and gold colloids before removing the cells and observed the specimens by the SE-ADM system. The 60-nm gold colloids were attached to the dense black structures in the cell detachment region (indicated by red and white arrows in Fig 3F and 3G). The high-density structures exhibit a smooth surface with no filament-like structures (Fig 3F and 3G). The 3D color map clearly shows the 60-nm gold colloids bound to the high-density particles (Fig 3H). These results strongly suggest that the high-density particles in the cell detachment region were focal adhesion cores containing integrin β1 without actin filaments.

**Fig 4:**
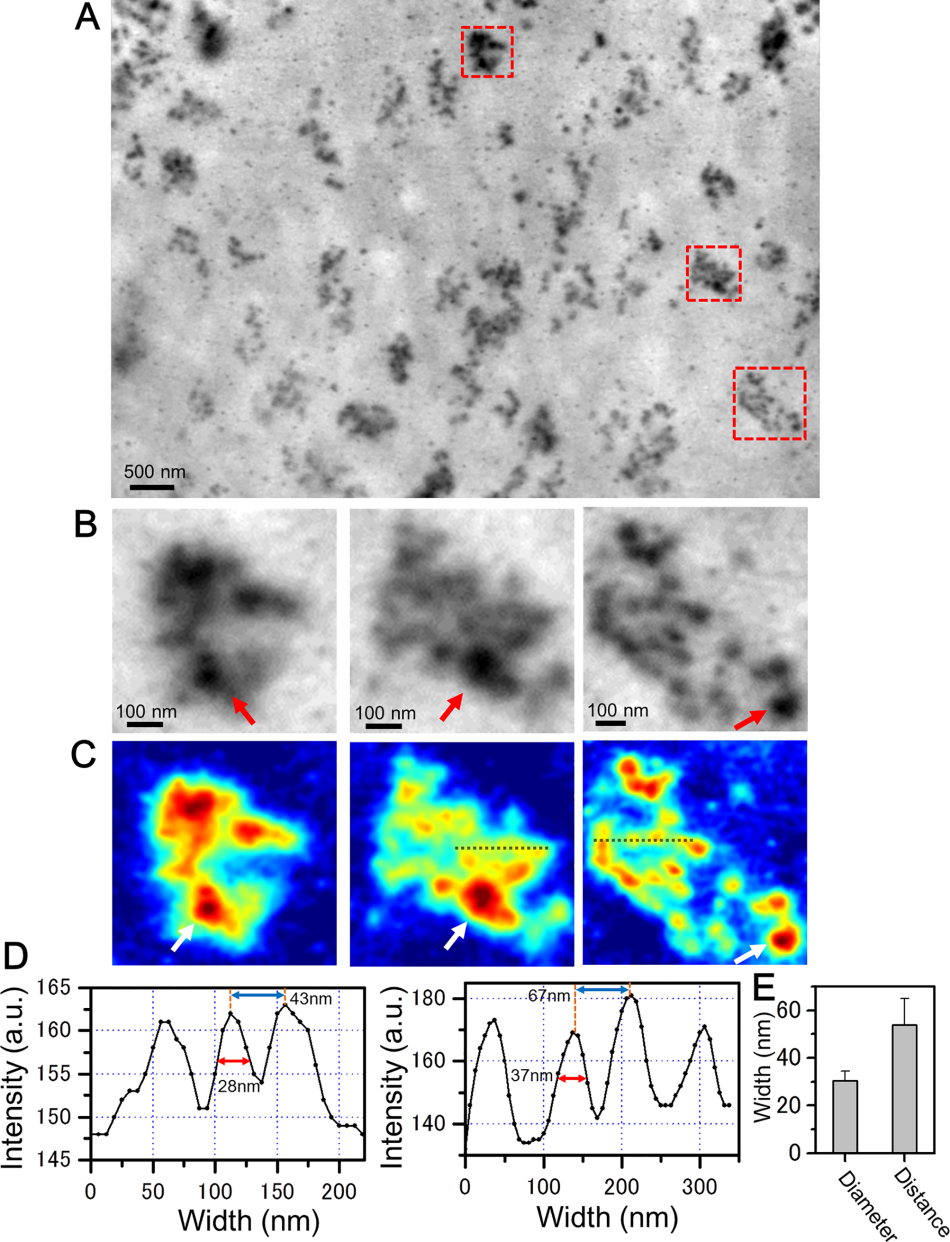
SE-ADM images of integrin granules bound to 60-nm gold colloids after cell detachment. (A) Dielectric impedance image of the cell adhesion core bound to 60-nm gold colloids via anti-integrin β1 antibody after cell removal (15,000× magnification, 6 kV electron beam). Adhesion cores consisting of small granules are dispersed throughout the imaging area. (B) Three enlarged images of the red-square region in (A). Red arrows indicate the 60-nm gold colloids. The adhesion cores clearly contain clusters of small granules with 60-nm gold colloids bound by anti-integrin β1 antibody. (C) Pseudo-color maps of (B) after intensity inversion. White arrows indicate the 60-nm gold colloids. Adhesion cores containing clusters of small granules are clearly visible. (D) Line plots of the integrin granular regions along the dotted lines in (C). (E) Mean diameter and separation of the granules in eight selected adhesion cores in panel (D) and S7 Fig (30.4 ± 4.0 and 53.9 ± 11.1 nm, respectively). Scale bars: 500 nm in (A) and 100 nm in (B).

To further analyze the adhesion core containing integrin β1, we observed other regions after cell detachment at 15,000× magnification (Fig 4). After removing the cells bound by the anti-integrin β1 antibody and 60-nm gold colloids, many granule-containing adhesion cores appeared across the scanned area (Fig 4A). For a precise analysis, the adhesion cores within the three red squares in Fig 4A were enlarged and intensity-inverted to pseudo-color maps (Fig 4B and 4C). The adhesion cores after cell detachment consisted of 10–20 small granules with 60-nm gold colloids. Line plots of the granular clusters (along the dashed lines in Fig 4C) exhibited three or four peaks (Fig 4D). We statistically calculated the diameters and separation distances of the integrin granules from eight selected adhesion cores imaged in Fig 4C and 4D and S7 Fig. The mean granular diameter and separation were 30.4 ± 4.0 and 53.9 ± 11.1 nm, respectively (Fig 4E). The granular diameter approximately corresponds to that of the integrin dimer structure analysed by AFM [12]. Moreover, the integrins in the adhesion core appeared to consist of 10–20 dimers.

**Fig 5:**
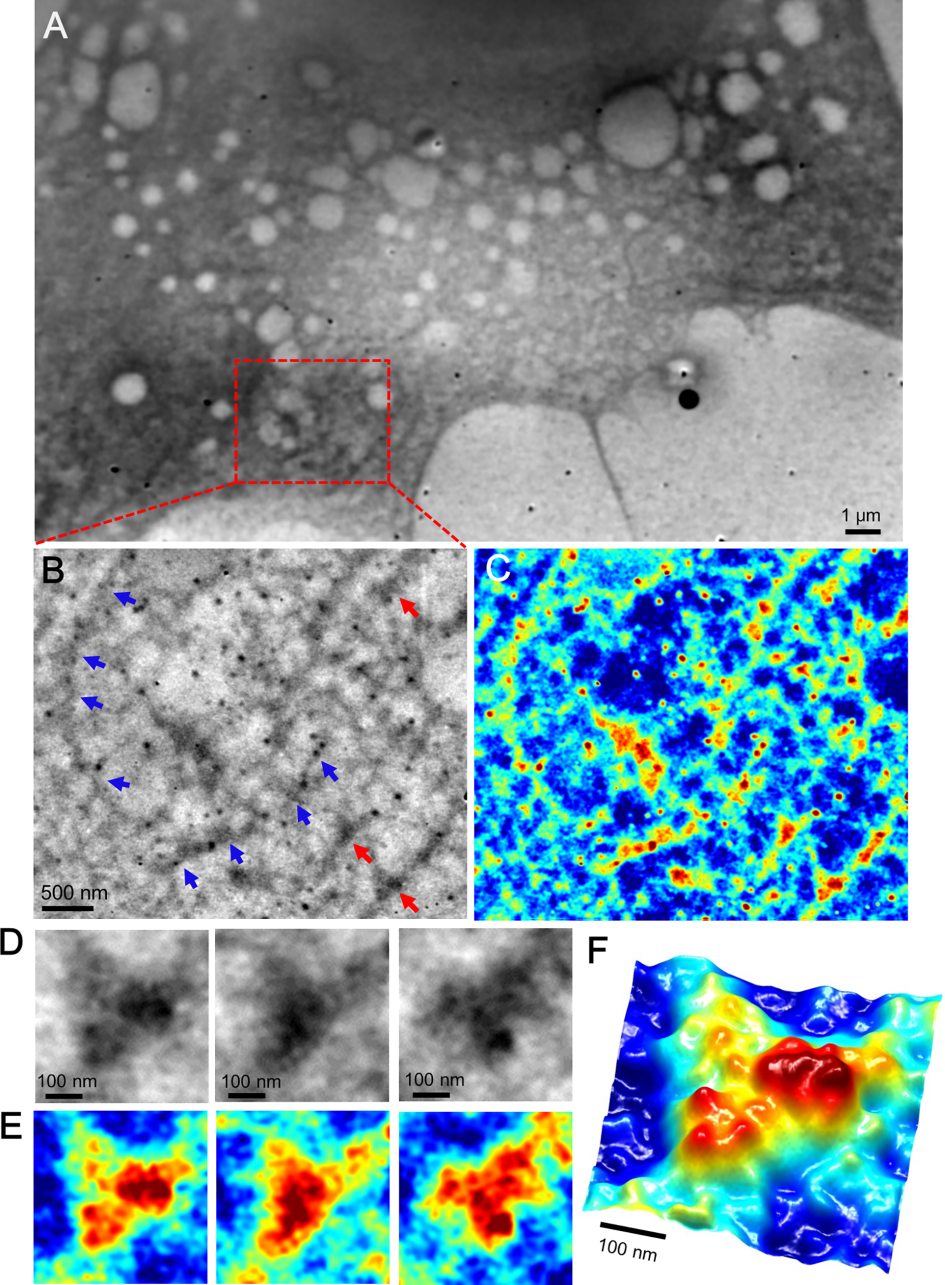
Images of F-actin in cancer cells stained with anti-F-actin antibody plus 60-nm gold colloids. (A) Dielectric impedance image of cells stained with anti-F-actin antibody and 60-nm gold colloids after paraformaldehyde fixation and 0.1% Triton-X permeabilization (3,000× magnification, 5 kV electron beam, −9 V bias). Expanded image (B) and pseudo-color map (C) after intensity inversion of the red boxed area in (A) at 20,000× magnification. The 60-nm gold colloids clearly attach to the mesh filament structure. The blue and red arrows show the actin filaments and high-density cores, respectively. (D) Three enlarged images of the black granule clusters connected to F-actin, indicated by red arrows in (B). (E) Pseudo-color maps of (D) after intensity inversion. (F) 3D color map of the left image of (E). Scale bars: 1 μm in (A), 500 nm in (B and C) and 100 nm in (D and F).

Focal adhesion domains are known to consist of integrins and various cytoskeletal-associated proteins, mainly actin and talin, which are connected to integrins [10, 11]. To ascertain the integrin adhesion structure connected to actin (as a typical cytoskeletal protein), we observed the F-actin structure in the mammalian cancer cells after labeling with anti-F-actin antibody and 60-nm gold colloids conjugated to a secondary antibody, fixing with 4% paraformaldehyde, and permeabilizing with 0.1% Triton-X (Fig 5A–5C). Fig 5A shows the whole-cell imaging by our SE-ADM system. Mesh-like filament structures with 60-nm gold colloids bound to F-actin are clearly visible in the cytoplasmic region (Fig 5B, blue arrows). As revealed in the intensity-inverted pseudo-color map, the 60-nm gold colloids were attached along the mesh-like structure of the actin filament (Fig 5C). Therefore, the mesh-like filament structure observed in Fig 1A and 1B was suggested to be the actin filament. Moreover, the high-intensity triangular and square-like cores (indicated by red arrows in Fig 5B) were confirmed to bind to actin filaments. The enlarged cores and their pseudo- and 3D pseudo-color maps are shown in Fig 5D–5F. Because the cells were fixed and permeabilized to stain the actin, the selected images (Fig 5D–5F) somewhat differed from the high-contrast cluster images in Fig 1C–1E, but can be identified as the same clusters. Similar mesh-like structures of actin fibers connected to adhesion cores were reported in previous studies [11].

**Fig 6:**
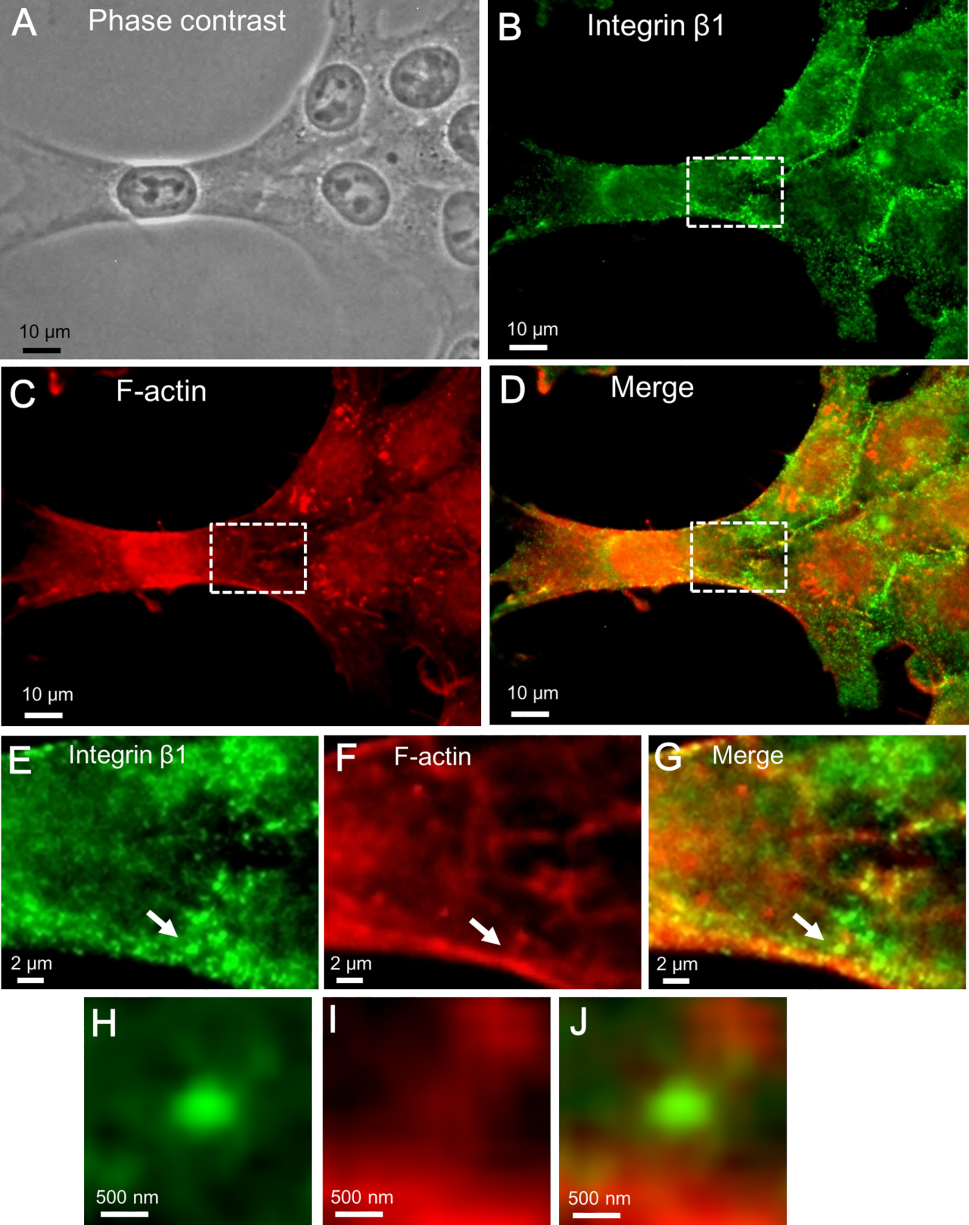
Optical phase contrast and fluorescence images of 4T1E/M3 cells stained with anti-integrin β1 and anti-F-actin antibodies. (A) Optical phase contrast image of cells double-stained with anti-integrin β1 and anti-F-actin antibodies after paraformaldehyde fixation and permeabilization (optical microscopy, 400× magnification). (B) Fluorescence image of anti-integrin β1 and FITC-immunostained cells under the optical microscope with a green fluorescence filter (400× magnification). (C) Fluorescence image stained with anti-F-actin antibody and rhodamine under the optical microscope with a red fluorescence filter (400× magnification). (D) Merged fluorescence image of the cells stained with anti-integrin β1 (B) and anti-F-actin antibodies (C). (E) and (F) Enlarged images of the areas enclosed by the white box in (B) and (C), respectively. (G) Merged fluorescence image of (E) and (F). (H) Enlarged image of an integrin β1 spot, indicated by the white arrow in (E). (I) Enlarged image of F-actin, indicated by the white arrow in (F). (J) Merged fluorescence image of integrin β1 (H) and F-actin (I). Scale bars: 10 μm in (A–D), 2 μm in (E–G), and 500 nm in (H–J).

**Fig 7:**
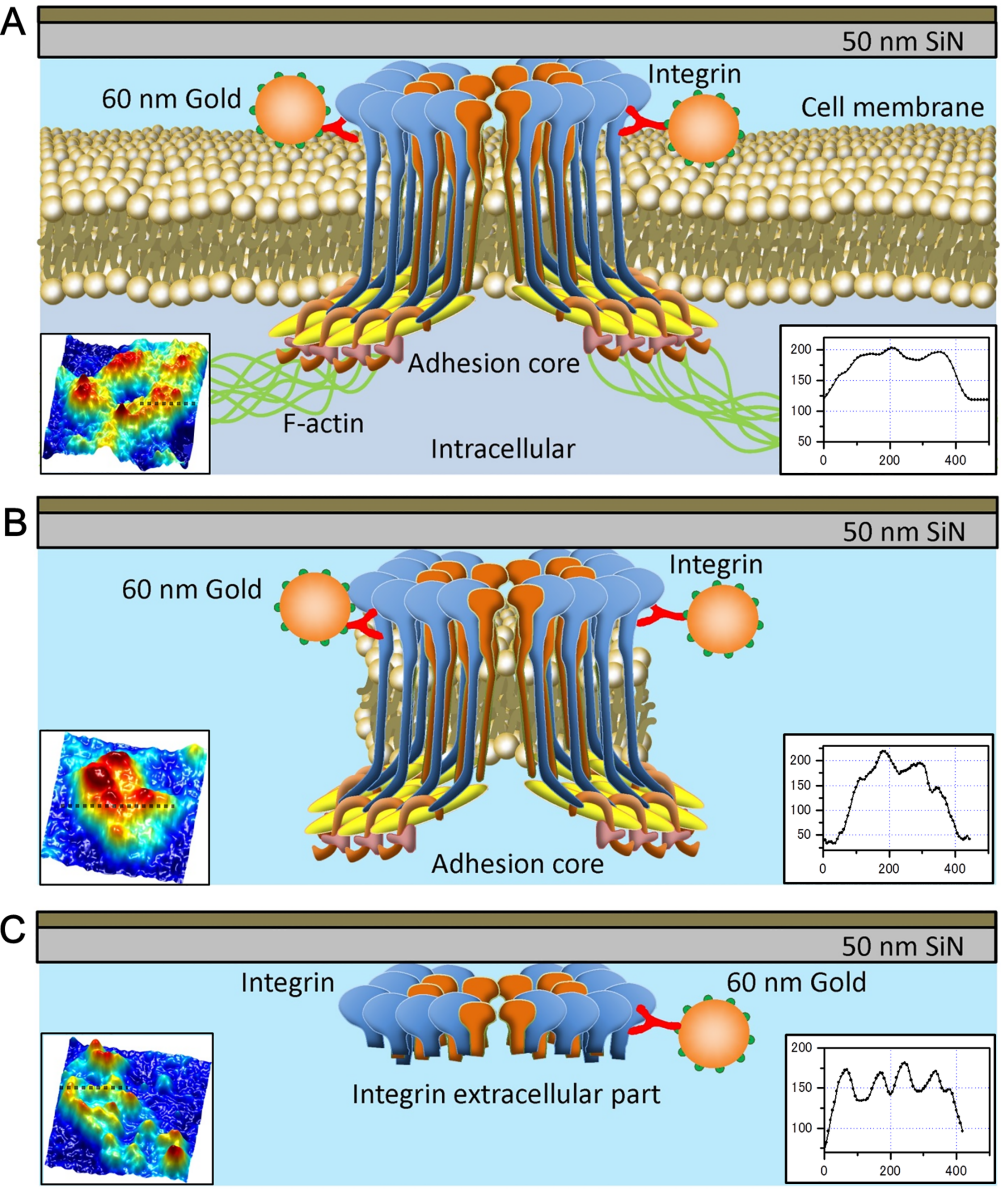
Schematic of structural organization of the focal adhesion core on the cell membrane. (A) Conceptual diagram of the focal adhesion core stained with streptavidin-conjugated 60-nm gold colloids and biotin-conjugated anti-integrin β1 antibody. The integrin adhesion core is connected to F-actin via related proteins such as FAK, talin, and vinculin. (B) When the integrins remain attached to other related proteins after cell removal, the adhesion cores are dense with high contrast. (C) When the integrin adhesion components remain alone on the silicon nitride film after cell removal, their adhesion cores contain granular clusters of approximate size of 30 nm (see the cross-sectional line plot, bottom-right inset). The mean particle diameter (determined from eight selected adhesion cores in the images of Fig 4C and 4D and S7 Fig) is 30.4 ± 4.0 nm. The bottom left insets in (A–C) present the corresponding 3D color maps of the adhesion core observed by the SE-ADM system, which were scanned along the dashed lines to obtain the cross-sectional plots. The vertical and horizontal axes of the line plot denote intensity and width, respectively.

Next, we analyzed fluorescence images of fixed, permeabilized 4T1E/M3 cells that were doubly stained with respective fluorescent secondary antibodies to the anti-integrin β1 and anti-F-actin antibodies. The nucleus was evident in the phase-contrast optical microscopic image of the doubly stained cells (Fig 6A). Fluorescent integrins on the cell membranes appeared as spots of various sizes (Fig 6B). By contrast, the F-actin fluorescence images revealed mesh-like filament structures in the cytoplasmic region (Fig 6C). The fluorescence images of integrin β1 and F-actin uncovered different patterns (Fig 6D). In the enlarged images of the cytoplasmic region (enclosed by the white square in Fig 6B–6D), the integrin adhesion core was found between the actin mesh structures (Fig 6E–6G). For a detailed analysis, we further enlarged the integrin spots (indicated by the white arrows in Fig 6E) and observed circular and ellipse fluorescence patterns of integrin β1 (Fig 6H). The same area of the F-actin image (Fig 6I) and its merged image (Fig 6J) indicate that the integrin adhesion cores were connected to the F-actin filaments. These results suggest that integrin adhesion cores connect to the actin mesh structures (Figs 2G and 7A). The line plot (Fig 7A, bottom-right inset) reveals that the adhesion cores peak at the locations of actin filaments. After removing the cells, these integrin-containing adhesion cores appeared to form two types of structures. When integrins attach to other related proteins and the partial membrane (Fig 7B), the adhesion cores are dense with high contrast and exhibit a smooth surface in the SE-ADM images (Fig 3H). The line plot also reveals a smooth adhesion-core peak without actin filaments. By contrast, when the integrins alone remained on the SiN film, the adhesion cores clustered into granules of approximate size of 30 nm (Figs 4C and 7C). Their line plots present integrin-dimer peaks without other adhesion proteins.

## Discussion

Integrins are adhesion receptors in focal adhesion cores that provide a physical link to the cytoskeleton of actin filaments [1, 2, 30]. The focal adhesion core consists of various proteins, such as integrins, talins, vinculin, and F-actin [10, 30]. Our SE-ADM system visually clarified the adhesion core on living intact cancer cells without staining and fixation (Fig 1A–1E). The adhesion core domain contains high-contrast triangular or square-like clusters with a few high-contrast, roughly surfaced granules (Fig 1E). Furthermore, the adhesion core is linked to mesh-like filament structures (Fig 1E). After staining with anti-integrin β1 antibodies and 60-nm gold colloids, the clear high-contrast clusters appeared to be the adhesion cores containing integrin β1 (Fig 2E–2G). Moreover, the mesh-like structure stained with 60-nm gold colloids and anti-F-actin antibody was suggested as the actin filament (Fig 5B and 5C).

When observed by fluorescence microscopy, the integrins appeared only as round particles (Fig 6H). By contrast, integrins targeted with the same anti-integrin β1 in SE-ADM revealed the characteristic granular structures of the integrin-containing adhesion cores (Fig 2E and 2F). Our SE-ADM also showed the mesh-like structure of the actin filaments (Fig 5B and 5C), which was partially observed but unclear in fluorescence microscopy (Fig 6C). Furthermore, our SE-ADM system simultaneously observed the 60-nm gold colloids and the binding intracellular structure with high contrast and high resolution (Figs 2 and 5), whereas conventional fluorescence microscopy detected only the fluorescence labels without the inner cell structures (Fig 6). Therefore, our system enables analyses of cell structures targeted by nanoparticle-tagged antibodies.

The intact adhesion cores on the cells contained high-contrast, roughly surfaced clusters attached by small granular particles and linked to the actin-filament structure (Figs 1C–1E, 2E–2G and 7A). When cells were removed from the SiN film, the remaining focal adhesion cores were clearly observed as smooth structures. The mesh-like filaments, suspected to be F-actin, were absent (Fig 3E and 3H). Within the smooth structures, we assumed that the integrin-containing adhesion core was attached to many constructing proteins, such as talin, FAK, and vinculin (Fig 7B). In other cell-detachment regions, we observed adhesion cores of approximate diameter of 30 nm, consisting of 10–20 small granules containing integrin β1 (Fig 4B–4E). In this case, the adhesion core may consist of integrins alone with no constructing proteins (Fig 7C). The diameter of the integrin heterodimers, measured by single-particle analysis of the EM protein images [17] and AFM [12, 13], was approximately 20–30 nm. In our images of intact living cells, the diameter of the adhesion core containing the integrin dimers was 30.4 ± 4.0 nm with a cycle of 53.9 ± 11.1 nm. These values were averaged over eight adhesion cores selected from Fig 4C–4E and S7 Fig. The hemidesmosomes of the cell slice image, observed by TEM, revealed 10–20 integrin dimers in the high-density clusters [14]. Furthermore, some hemidesmosomes are known to cluster at the adhesion region and attach to actin filaments [14], and actin networks are connected to many receptors in the cell membrane [31]. High-resolution images of actin mesh structures have been observed by TEM [32] and total internal reflection fluorescence microscopy [33, 34]. Our results are consistent with these previous findings.

Our SE-ADM system enables nanoscale imaging of living cells without metal staining or fixation [25]. In this study, we clearly detected the intact adhesion ultrastructure on the membranes of untreated cells. Recently, nanoscale images captured by advanced techniques have revealed the superstructure of focal-adhesion cores on cells [10, 11, 16]. Super-resolution fluorescence microscopy using an iPALM system with fluorescently labeled adhesion proteins has clarified the 3D structures of focal adhesion cores in living cells [10]. Super-resolution fluorescence microscopy (namely, SIM and dSTORM) has also detected the 2D structures of integrin and F-actin [11], but requires fluorescence labeling of the proteins. An AFM system and single-particle analysis have obtained high-resolution images and/or the 3D structures of single integrin heterodimers [12]. However, these analyses require extraction and purification of integrin molecules from the cells [12]. None of these techniques can visualize the integrin-containing adhesion cores on intact living cells. By contrast, our SE-ADM reveals the intact structures of adhesion cores on living cells without labeling or fixation (Fig 1).

To analyze the spatial resolution of the SE-ADM system, we investigated the metal-coated SiN film on an atmospheric holder in Monte Carlo simulations using CASINO Ver. 2.42 [35] (S8 Fig). Under a beam-accelerating voltage of 4 kV, almost all of the electrons passing through the W layer were absorbed by the SiN film (S8A Fig). The spatial resolution of the SE-ADM system depends on the absorbed-energy width in the SiN film (S8B Fig). Therefore, the spatial resolution could be improved by modifying the thin SiN film (S8C Fig).

At the present scanning time of our SE-ADM system (80 s per image), we cannot capture the high-speed motions of macromembrane proteins. The movement of integrin dimers on the cell membrane has recently been reported [36, 37]. To ascertain the movements of integrin adhesion cores under our experimental conditions, we acquired two integrin fluorescence images separated by an 80-s interval (S9 Fig). The two fluorescence images are very similar (S9A and S9B Fig). When the second image (80 s later) was subtracted from the first image, the integrin fluorescence almost disappeared (S9C Fig). The similarity of the two images is also revealed in magnified images of the center region (S9D–S9F Fig). The subtraction image of the first and second central regions was below 25 intensities, 1/10 that of the initial fluorescence image (S9G and S9H Fig). These results suggest that the integrin adhesion cores were highly immobilized under our experimental conditions (confluent cell monolayer at room temperature). Therefore, the scanning time (80 s) may be reasonable for observing the integrin adhesion cores on the cells. However, the quicker movements of macromolecules may blur the images obtained by our current SE-ADM system. To detect the movements of membrane proteins, we should decrease the scanning time to nearly the video frame rate. Such a system might be realized by a low-noise cooling amplifier and resistance elements. In our future work, we will install a high-speed pre-amplifier with a wide frequency range pre-amplifier, which will reduce the imaging time to a few seconds.

Our new system is expected to observe various living cell movements, such as exocytosis of endoplasmic reticula and microtubule extension, in future researches. This achievement would further expand the new field of true visualization of macromolecules and their motions in living cells.

## Conclusion

We directly observed adhesion cores containing integrin β1 on mammalian cancer cells cultured in medium. Moreover, we clearly detected 60-nm gold colloids bound to integrin β1 via an antibody. The adhesion core contains three or four dense regions containing integrin β1 and connects to the F-actin filaments in intact cells. A high-density adhesion core may contain 10–20 integrin dimers. Our methods can also observe various other membrane proteins of intact cells in medium.

## Materials and methods

### 4T1E/M3 cell culture and sample preparation

Mouse breast cancer cells (4T1E/M3) were established as previously described [27–29]. Cells were cultured in high-glucose RPMI-1640 medium containing 10% fetal calf serum and 20 mM HEPES at 37°C under 5% CO_2_. After adding the medium (1.5 mL/dish) to a culture dish attached to a 50-nm SiN–Al holder, the cells (4 × 10^4^; 20 μL/dish) were seeded and cultured at 37°C under 5% CO_2_. The medium was changed after 2–3 days, and the cells formed a sub-confluent or completely confluent monolayer on the SiN membrane in the holder after 4–5 days [25].

### Immunolabelling of cells using antibody and 60-nm gold colloids

The cells seeded in the dish holder on 50-nm SiN film were stained with biotin-conjugated anti-mouse integrin β1 antibody (ABCAM, Cambridge, UK, catalogue #: ab23825, 1/100) diluted in a 1:1 mixture of PBS and medium (50 μL) for 30 min at 4°C or room temperature (r.t.), washed twice with the mixture solution and stained with streptavidin-conjugated 60-nm gold colloids (Cytodiagnostics Inc. Ontario, Canada, catalogue #: AC-60-04, 1/50) diluted in a 1:1 mixture of PBS and medium (50 μL) for 30 min at 4°C or r.t. After washing twice, the holder was attached to the acrylic sample holder in the 50-nm SiN film and observed using the SE-ADM system [25].

For the staining of F-actin, cells were fixed with 4% paraformaldehyde diluted in PBS for 10 min at r.t. and washed twice. Then the cells were stained with rabbit anti-mouse F-actin antibody (Bioss Inc., Massachusetts, USA, catalogue #: bs-1571R, 50 μL, 1/100) diluted in PBS including 0.1% Triton-X for permeabilisation for 30 min at r.t., washed twice with PBS and stained with anti-rabbit IgG-conjugated 60-nm gold colloids (Cytodiagnostics Inc., Ontario, Canada, catalogue #: AC-60-17, 1/100) diluted in PBS (50 μL) for 30 min at r.t. After washing twice, the dish was observed using the SE-ADM system.

We performed control experiments without anti-integrin antibody or anti-actin antibody and confirmed that there are little nonspecific gold-colloid binding (S4 Fig).

### Immunofluorescence staining of the cells and observation by optical microscopy

Cells seeded in a 35-mm glass-bottom dish (Matsunami Glass Ind., Ltd., Osaka, Japan) were stained with biotin-conjugated anti-mouse integrin β1 antibody (ABCAM, 1/100) diluted in a 1:1 mixture of PBS and medium (200 μL) for 30 min at 4°C, washed twice with the mixture solution and stained with streptavidin-conjugated FITC (Vector Laboratories, Inc., California, USA, catalogue #: A-2001, 1/100) diluted in a 1:1 mixture of PBS and medium (200 μL) for 30 min at 4°C. After washing twice, the holder was observed under a fluorescence optical microscope.

For double fluorescence staining of integrin β1 and F-actin, 4T1E/M3 cells seeded in a glass-bottom dish were fixed in 4% paraformaldehyde diluted in PBS for 10 min at r.t. and washed twice. Then, the cells were stained with biotin-conjugated anti-mouse integrin β1 antibody (ABCAM, 1/100) diluted in a 1:1 mixture of PBS and medium (200 μL) for 30 min at r.t., washed twice with the mixture solution and stained with streptavidin-conjugated FITC fluorescence (Vector Laboratories, Inc., California, USA, catalogue #: A-2001, 1/100) at r.t. for 30 min. Next, these cells were stained with rabbit anti-mouse F-actin antibody (Bioss Inc., Massachusetts, USA, catalogue #: bs-1571R, 1/100) diluted in a 1:1 mixture of PBS and medium (200 μL) including 0.1% Triton-X for permeabilisation for 30 min at r.t., washed twice with the mixture solution and stained with anti-rabbit IgG-conjugated rhodamine (Jackson Immunoresearch Laboratory Inc., Pennsylvania, USA, catalogue #: 711-065-152, 1/100) diluted in a 1:1 mixture of PBS and medium (200 μL) for 30 min at r.t. After washing twice, the dish was observed under a fluorescence optical microscope.

Cultured 4T1E/M3 cells stained for immunofluorescence analysis in a 35-mm-diameter glass-bottom dish (Matsunami Glass Ind., Japan) were visualised at 400× magnification using an optical phase microscope (AXIO Observer A1; Carl Zeiss, Oberkochen, Germany). Fluorescent images of the cancer cells were observed using a fluorescence filter with excitation/emission wavelengths of 480/520 nm (green fluorescence) and 565/620 nm (red fluorescence).

### Liquid sample and culture dish holders

The liquid sample holder was formed as previously described [25]. Briefly, the liquid sample holder comprised an upper Al holder and lower acrylic resin portion that maintained the sample solution at atmospheric pressure between the SiN films [24, 25]. A 50-nm-thick SiN film supported by a 0.4 × 0.4 mm^2^ window in a Si frame (4 × 4 mm^2^, 0.38 mm thickness; Silson Ltd., Warwickshire, UK) was coated with W using a magnetron sputtering device (Model MSP-30T, Vacuum Device Inc., Ibaraki, Japan), as previously described [24]. The upper W-coated SiN film was attached to the Al holder with double-sided tape, and the W layer on the SiN film was connected to the Al holder using silver conductive ink. A hand-made Al holder with a Si frame was attached under a 35-mm culture dish-sized square hole in the centre with double-sided tape, as previously described [25]. The culture dish holder was subsequently UV-sterilised for 17–18 h.

4T1E/M3 mouse breast cancer cells were cultured in the holder dish and stained with biotin-conjugated anti-mouse-integrin β1 antibody and streptavidin-conjugated 60-nm gold colloids as described previously. Next, the cell-containing Al holder was separated from the plastic culture dish, attached upside down to another SiN film on an acrylic plate and sealed [25]. The Al holder received a voltage bias of approximately −9 V (S1 Fig).

### Cell detachment from SiN film

The 4T1E/M3 cells formed a sub-confluent or complete confluent monolayer on the SiN membrane in the holder after 4–5 days. The cell monolayers were softly detached from the SiN film by opening the dish holder after once setting the acrylic holder. After removing the cells, the dish holder was attached to a new acrylic holder again, and the dish holder set was installed into the SE-ADM system (S5 Fig).

### High-resolution SE-ADM system and FE-SEM setup

The FE-SEM (JSM-7000F, JEOL, Tokyo, Japan) based high-resolution SE-ADM imaging system is shown in S1 Fig. The liquid sample holder was mounted onto the SEM stage, and the detector terminal was connected to a pre-amplifier under the holder [24]. The electrical signal from the pre-amplifier was fed into the AD converter (AIO-163202FX-USB, Contec Co. Ltd., Osaka, Japan) after low-pass filtering, as previously described [24]. The LPF and electron beam-scan signals were logged using a PC through an AD converter at a sampling frequency of 50 kHz. SEM images (1,280 × 1,020 pixels) were captured at 1,500–20,000× magnification with a scanning time of 80 s, working distance of 7 mm, EB acceleration voltage of 4–8 kV and current of 10 pA.

### Image processing

SE-ADM signal data from the AD converter were transferred to a personal computer (Intel Core i7, 2.8 GHz, Windows 7), and high-resolution SE-ADM images were processed from the LPF signal and scanning signal using the image-processing toolbox of MATLAB R2014a (Math Works Inc., Natick, MA, USA). The original SE-ADM images were filtered using a 2D Gaussian filter (GF) with a kernel size of 11 × 11 pixels and a radius of 1.2σ. Background subtraction was achieved by subtracting SE-ADM images from the filtered images using a broad GF (400 × 400 pixels, 200σ).

### Monte Carlo simulation

Electron trajectories in the W-coated SiN film were calculated by Monte Carlo simulations using CASINO version 2.42 [35]. The density and thickness were 19.3 g/cm^3^ and 15 nm, respectively, in the W layer and 3.12 g/cm^3^ and 10–70 nm, respectively, in the SiN film. The simulation parameters were as follows: 100,000 electrons, EB accelerating voltages = 4 kV, and EB spot diameter = 3 nm. The simulations were performed on a personal computer (Intel Core i7-3930K, 3.2 GHz, Windows 7).

## Supporting information

S1 Fig Schematic of the experimental setup in the scanning electron-assisted dielectric microscopy (SE-ADM) system based on FE-SEM. Mammalian cancer cells were cultured in liquid medium inserted between two silicon nitride (SiN) films in the liquid holder. The scanning electron beam (EB) was aimed at the upper tungsten (W)–SiN film. The measurement terminal under the holder detects the electrical signal from the irradiated part of the W–SiN film. The electrical signal is converted to a digital signal by an AD converter after DC pre-amplification. The SE-ADM images were constructed from the electrical signal and the EB scanning signal using MATLAB R2014a software. The bias voltage on the W layer was −9 V.
(TIF)

S2 Fig Integrin β1 fluorescence image of mammalian cancer cells. (A) Optical phase contrast image of cells stained with anti-integrin β1 antibody. The cells were stained with rabbit anti-integrin β1 antibody and FITC-conjugated anti-rabbit IgG and observed by optical microscopy at 400× magnification. (B) Green-filtered fluorescence image of (A) at 400× magnification. (C) Enlarged image of the integrin β1 spots within the red square in (B), showing that 4T1E/M3 cells strongly express integrin β1. (D) Optical phase contrast image of the detachment-cell region after anti-integrin β1 immunostaining. Small granules are dispersed throughout the region. (E) Integrin β1 fluorescence image of the integrin β1 bound to the glass bottom after cell detachment. (F) Enlarged image of the integrin β1 spots within the red square in (E). Scale bars: 10 μm in (A–B) and (D–E), 1 μm in (C), and 2 μm in (F).
(TIF)

S3 Fig SE-ADM image of the 60-nm gold colloids. (A) and (B): Two dielectric images of streptavidin-conjugated 60-nm gold colloids in liquid (50,000× magnification, 4 kV electron beam acceleration). The 60-nm gold colloids appear as distinct black spheres. Both scale bars are 100 nm.
(TIF)

S4 Fig SE-ADM image of the adhesion core of 4T1E/M3 cells stained by streptavidin-conjugated 60-nm gold colloid without anti-integrin antibody. (A) Dielectric image of 4T1E/M3 cells stained by streptavidin-conjugated 60-nm gold colloids in medium (10,000× magnification, 6 kV electron beam, −9 V bias). (B) Another image of the same specimen in a different region (10,000× magnification, 8 kV electron beam, −9 V bias). (C) Three enlarged images of the adhesion cores indicated by the red arrows in (A) and (B) showing clear adhesion cores without gold colloids. (D) 3D color map of the left side of (C). Scale bars: 1 μm in (A–B) and 200 nm in (C).
(TIF)

S5 Fig Schematic of soft cell removal from the silicon nitride (SiN) film. (A) The Al holder covered with tungsten (W)-coated SiN film was attached at the bottom of the culture dish, and cells and medium were added. After 4–5 days of culture, the cancer cells formed a confluent monolayer in the holder. The cell-containing Al holder was separated from the plastic culture dish (B) and attached upside down to another SiN film on an acrylic plate (C) (enlarged to show the cells in C′). (D) The Al holder was separated from the acrylic plate, and the cells were detached from the upper W-coated SiN film, leaving the adhesion cores alone. (E) and (F) The dish holder with the adhesion cores was attached to a new acrylic holder and re-installed in the SE-ADM system.
(TIF)

S6 Fig Focal adhesion cores after cell removal. (A–F) Enlargements of six adhesion cores after cell removal, observed by the SE-ADM system (10,000× magnification, 7 kV EB, 7 mm working distance, −9 V bias). The left and central panels show the enlarged images and their intensity-inverted pseudo-color maps, respectively. The right panels are the line plots along the dotted lines of the adhesion core regions in the corresponding pseudo-color maps. The diameter of the adhesion core (430 ± 56.1 nm) was averaged over nine adhesion cores selected from these images and those in Fig 3. All scale bars are 200 nm.
(TIF)

S7 Fig Focal adhesion cores of integrin granules bound to 60-nm gold colloids after cell removal. (A–F) Enlargements of six adhesion cores containing small granules observed by the SE-ADM system (15,000× magnification, 6-kV EB acceleration, 7 mm working distance, −9 V bias). The left and central panels show the enlarged images and their intensity-inverted pseudo-color maps, respectively. The right panels are the line plots of the integrin granular regions along the dotted lines in the corresponding pseudo-color maps. The diameter and separation of the adhesion particles (30.4 ± 4.0 and 53.9 ± 11.1 nm, respectively) were averaged over eight adhesion cores selected from these images and those in Fig 4. All scale bars are 100 nm.
(TIF)

S8 Fig Monte Carlo simulation of metal-coated SiN film on an atmospheric holder. (A) Monte Carlo simulation of the W-coated SiN film executed in CASINO ver. 2.42 [35], showing the electron trajectory area of the 50-nm-thick SiN film on the 15-nm-thick W layer. The EB spot diameter was 3 nm. At a beam-accelerating voltage of 4 kV, almost all of the electrons passing through the W layer were absorbed by the SiN film. (B) Cross-section of the energy deposited in the W-coated SiN film by the 4 kV electrons in the EB, normalized by each layer of the film. (C) Full-width-at-half-maximum of the energy absorbed in the SiN film as a function of film thickness (10–70 nm). The acceleration voltage and W layer thickness were fixed at 4 kV and 15 nm, respectively. Plotted are the averages and standard deviations of five simulations at each setting. The spatial resolution of the SE-ADM system depends on the width of the absorbed energy in the SiN film.
(TIF)

S9 Fig Comparison of integrin adhesion cores in an initial (time = 0) fluorescence image and a later (time = 80 s) fluorescence image. (A) Initial fluorescence image of 4T1E/M3 cells stained with biotin-conjugated rabbit anti-integrin β1 antibody and FITC-conjugated streptavidin and observed by an optical fluorescence microscope at 400× magnification with a green fluorescence filter. (B) Fluorescence image of the same region after 80 s. (C) Intensity image of the initial minus the later image. The integrin structure has almost completely disappeared. (D), (E), and (F) Enlarged images of the integrin β1 spots enclosed in the red squares in (A), (B), and (C), respectively, implying that the adhesion cores are almost immobilized under our experimental conditions. (G) Image-intensity distributions of the initial (black) and subtraction (red) images. The intensity of the subtraction image is below 25 at almost all locations. (H) Ratios of summation intensities between the initial and later images (black bar) and between the initial and subtraction images (red bar). The mean ratios, determined from 10 images, are 1.014 ± 0.0425 and 0.096 ± 0.011, respectively. The fluorescence intensities of the first and second images are almost identical (the subtraction image is 1/10 as intense as the initial image). Scale bars: 20 μm in (A–C) and 5 μm in (D–F).
(TIF)
