# Supplementary figures and images for: Nanoscale imaging of the adhesion core including integrin β1 on intact living cells using scanning electron-assisted dielectric-impedance microscopy
Source: PLoS One. 2018 Sep 20;13(9):e0204133. doi: 10.1371/journal.pone.0204133 (PMC6147470; doi:10.1371/journal.pone.0204133)

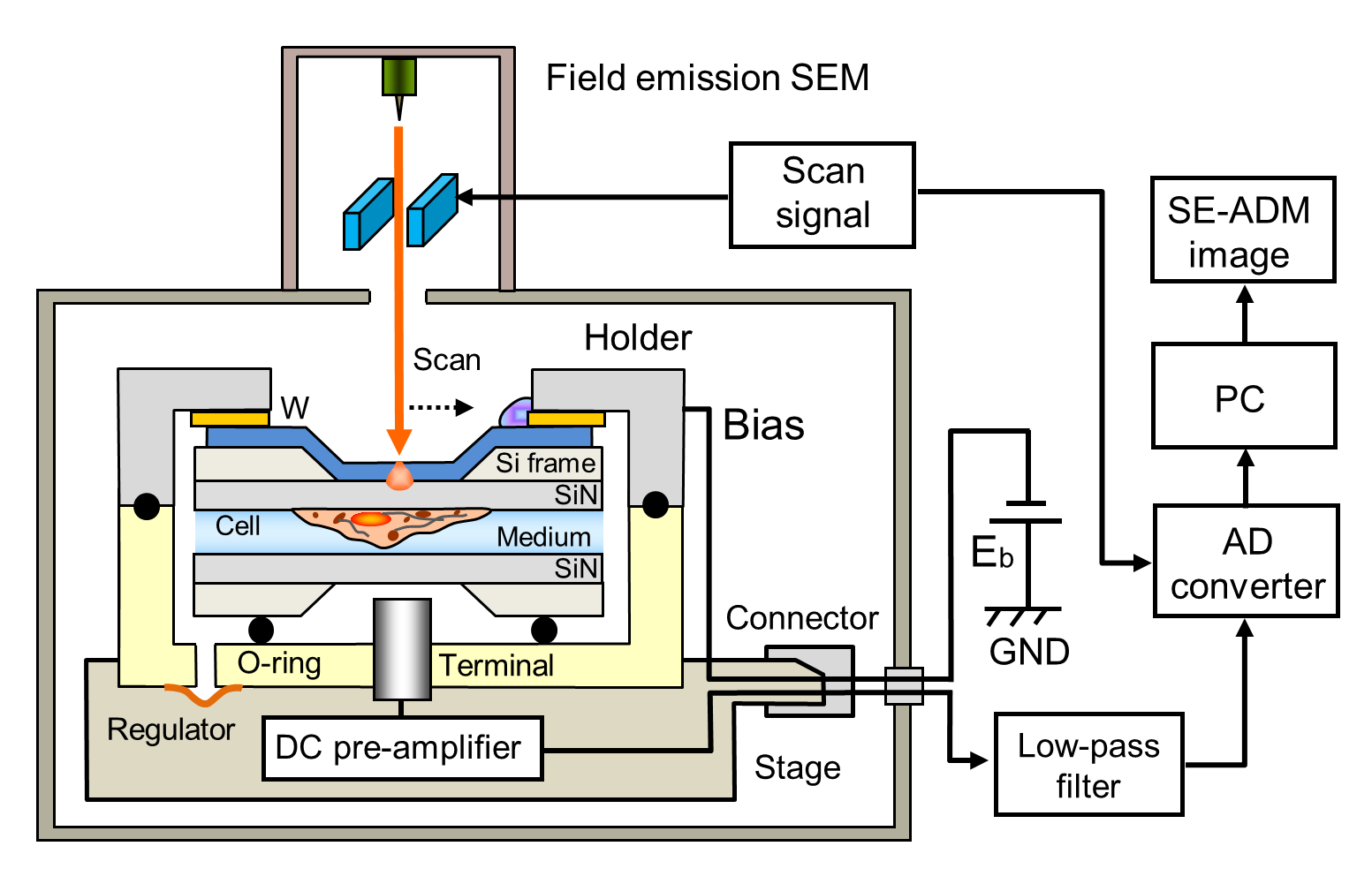

Supplement: S1 Fig — Mammalian cancer cells were cultured in liquid medium inserted between two silicon nitride (SiN) films in the liquid holder. The scanning electron beam (EB) was aimed at the upper tungsten (W)–SiN film. The measurement terminal under the holder detects the electrical signal from the irradiated part of the W–SiN film. The electrical signal is converted to a digital signal by an AD converter after DC pre-amplification. The SE-ADM images were constructed from the electrical signal and the EB scanning signal using MATLAB R2014a software. The bias voltage on the W layer was −9 V. (TIF) [file pone.0204133.s001.tif]

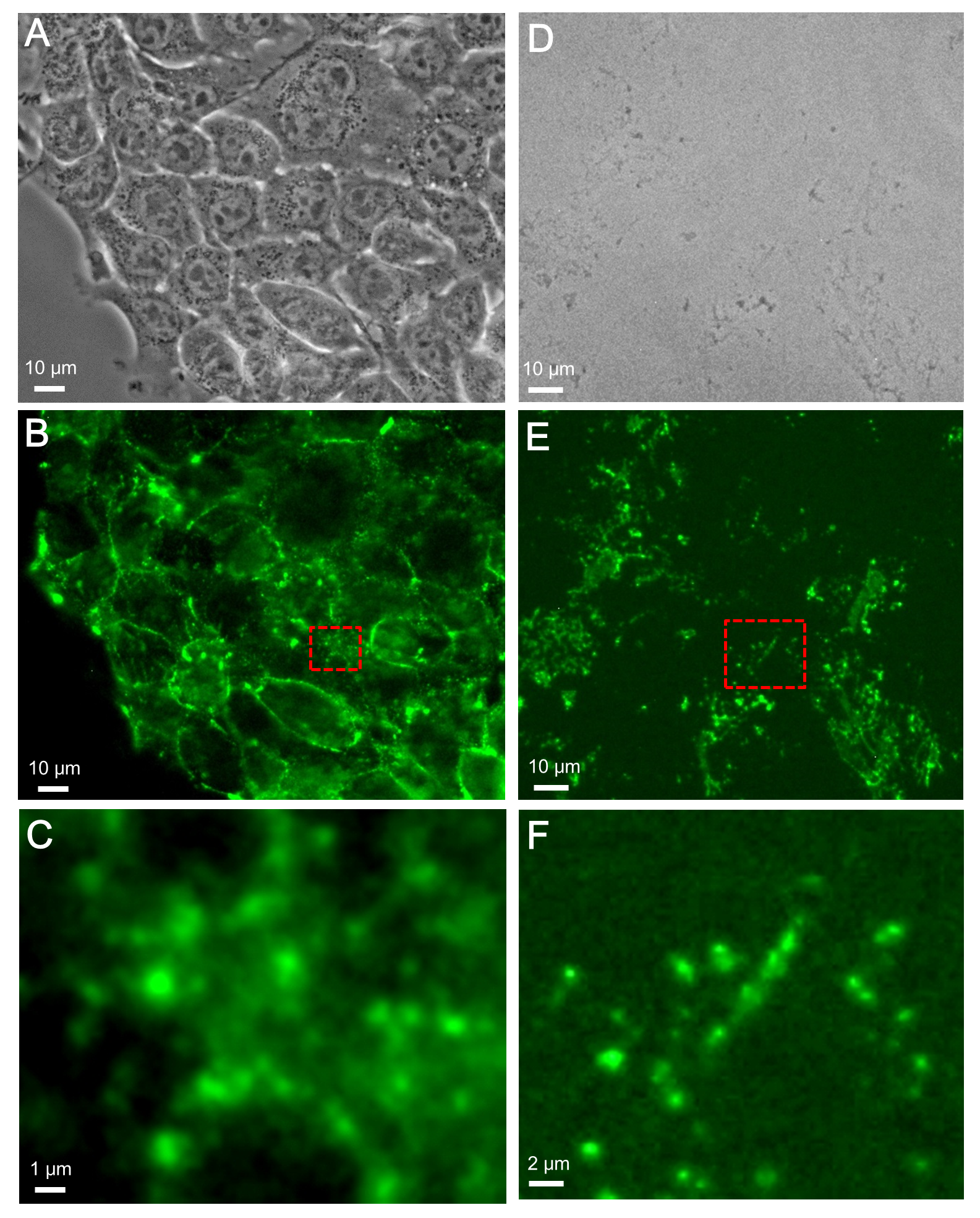

Supplement: S2 Fig — (A) Optical phase contrast image of cells stained with anti-integrin β1 antibody. The cells were stained with rabbit anti-integrin β1 antibody and FITC-conjugated anti-rabbit IgG and observed by optical microscopy at 400× magnification. (B) Green-filtered fluorescence image of (A) at 400× magnification. (C) Enlarged image of the integrin β1 spots within the red square in (B), showing that 4T1E/M3 cells strongly express integrin β1. (D) Optical phase contrast image of the detachment-cell region after anti-integrin β1 immunostaining. Small granules are dispersed throughout the region. (E) Integrin β1 fluorescence image of the integrin β1 bound to the glass bottom after cell detachment. (F) Enlarged image of the integrin β1 spots within the red square in (E). Scale bars: 10 μm in (A–B) and (D–E), 1 μm in (C), and 2 μm in (F). (TIF) [file pone.0204133.s002.tif]

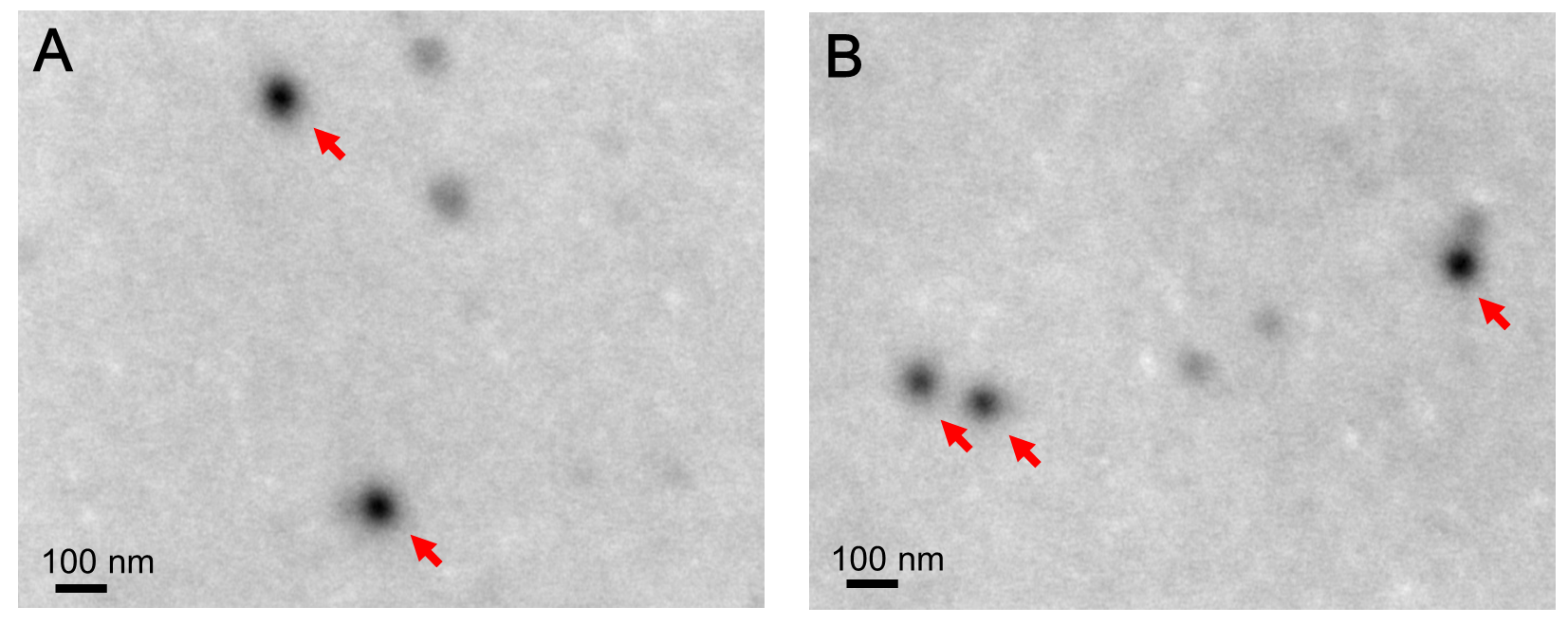

Supplement: S3 Fig — (A) and (B): Two dielectric images of streptavidin-conjugated 60-nm gold colloids in liquid (50,000× magnification, 4 kV electron beam acceleration). The 60-nm gold colloids appear as distinct black spheres. Both scale bars are 100 nm. (TIF) [file pone.0204133.s003.tif]

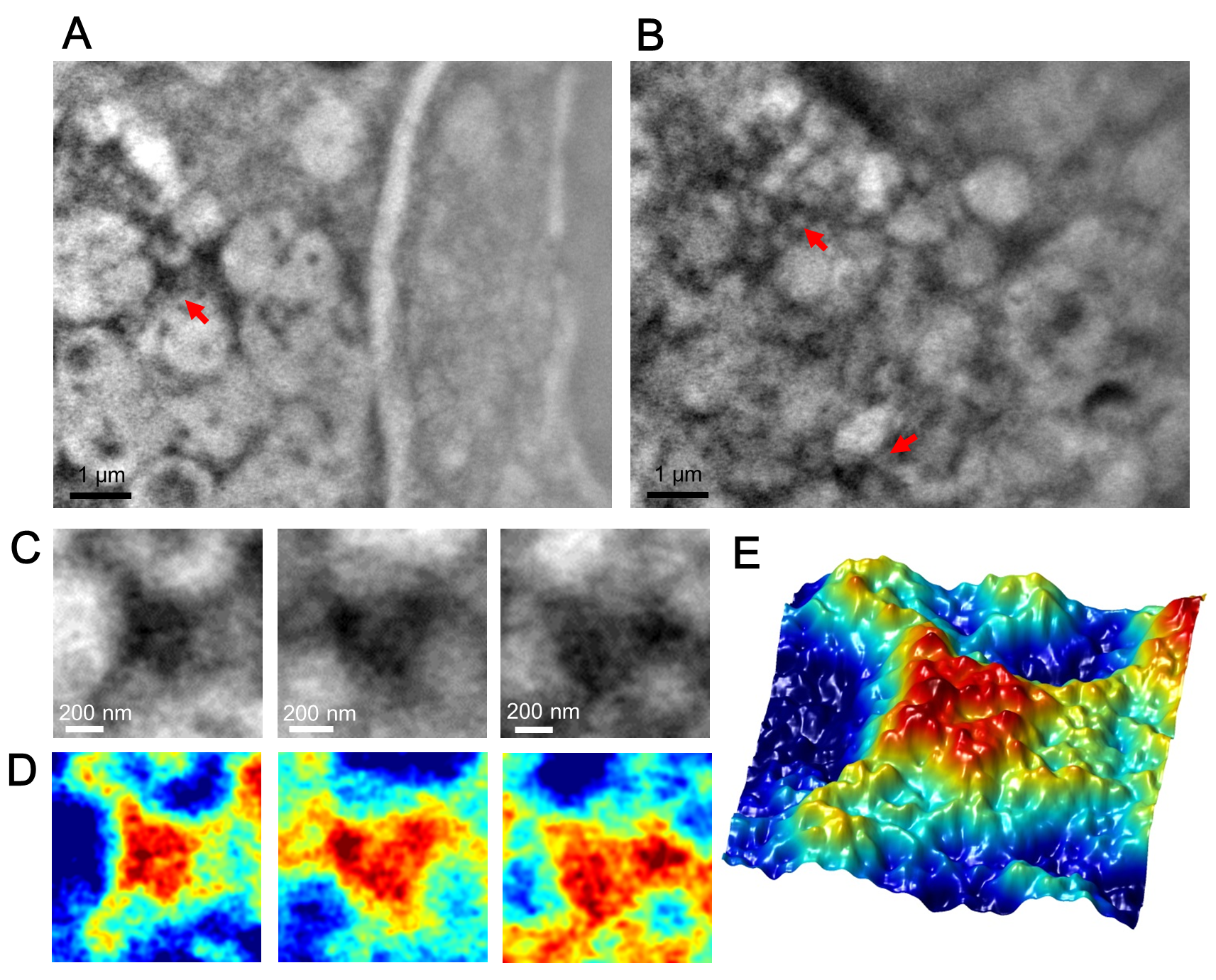

Supplement: S4 Fig — (A) Dielectric image of 4T1E/M3 cells stained by streptavidin-conjugated 60-nm gold colloids in medium (10,000× magnification, 6 kV electron beam, −9 V bias). (B) Another image of the same specimen in a different region (10,000× magnification, 8 kV electron beam, −9 V bias). (C) Three enlarged images of the adhesion cores indicated by the red arrows in (A) and (B) showing clear adhesion cores without gold colloids. (D) 3D color map of the left side of (C). Scale bars: 1 μm in (A–B) and 200 nm in (C). (TIF) [file pone.0204133.s004.tif]

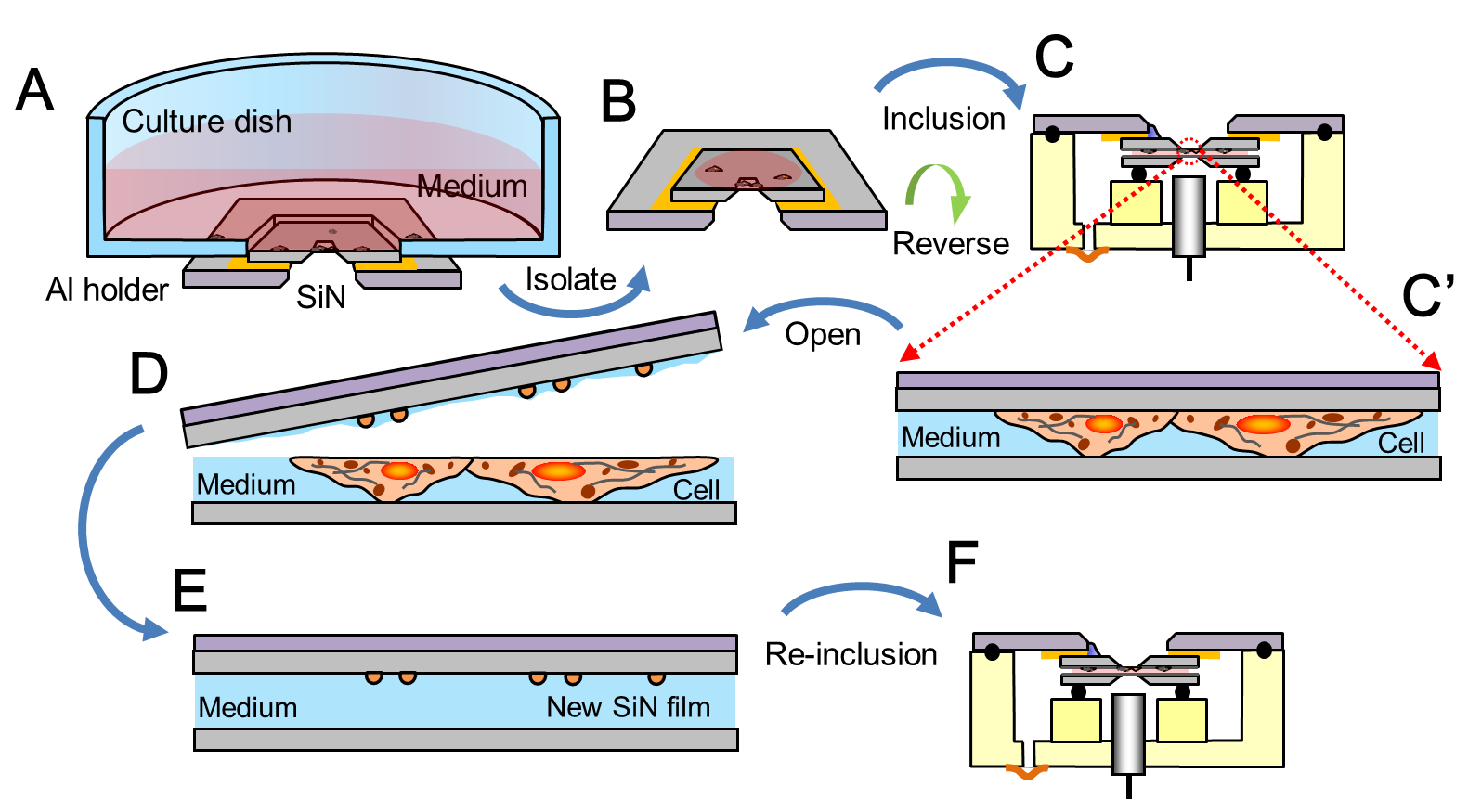

Supplement: S5 Fig — (A) The Al holder covered with tungsten (W)-coated SiN film was attached at the bottom of the culture dish, and cells and medium were added. After 4–5 days of culture, the cancer cells formed a confluent monolayer in the holder. The cell-containing Al holder was separated from the plastic culture dish (B) and attached upside down to another SiN film on an acrylic plate (C) (enlarged to show the cells in C′). (D) The Al holder was separated from the acrylic plate, and the cells were detached from the upper W-coated SiN film, leaving the adhesion cores alone. (E) and (F) The dish holder with the adhesion cores was attached to a new acrylic holder and re-installed in the SE-ADM system. (TIF) [file pone.0204133.s005.tif]

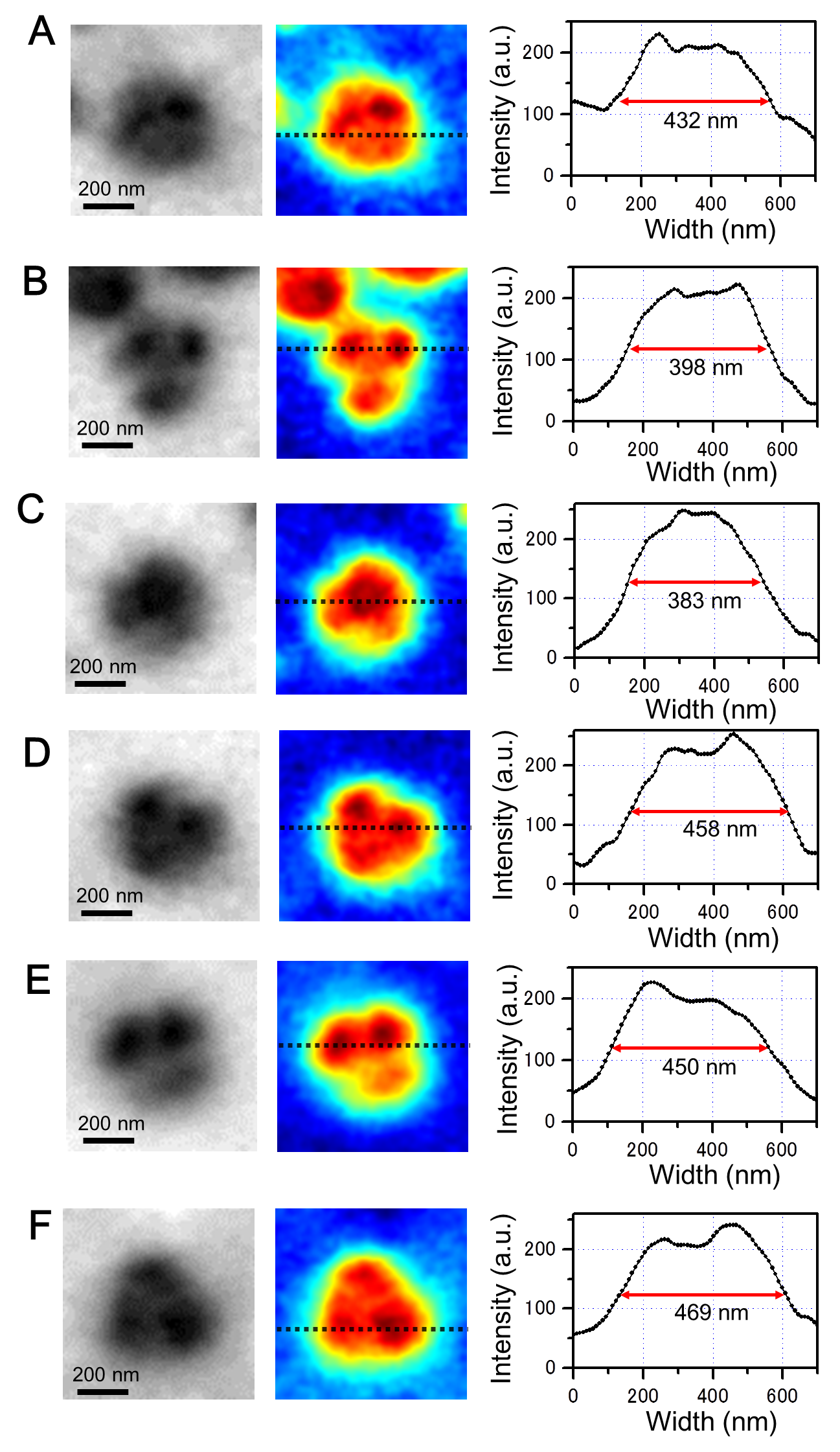

Supplement: S6 Fig — (A–F) Enlargements of six adhesion cores after cell removal, observed by the SE-ADM system (10,000× magnification, 7 kV EB, 7 mm working distance, −9 V bias). The left and central panels show the enlarged images and their intensity-inverted pseudo-color maps, respectively. The right panels are the line plots along the dotted lines of the adhesion core regions in the corresponding pseudo-color maps. The diameter of the adhesion core (430 ± 56.1 nm) was averaged over nine adhesion cores selected from these images and those in Fig 3. All scale bars are 200 nm. (TIF) [file pone.0204133.s006.tif]

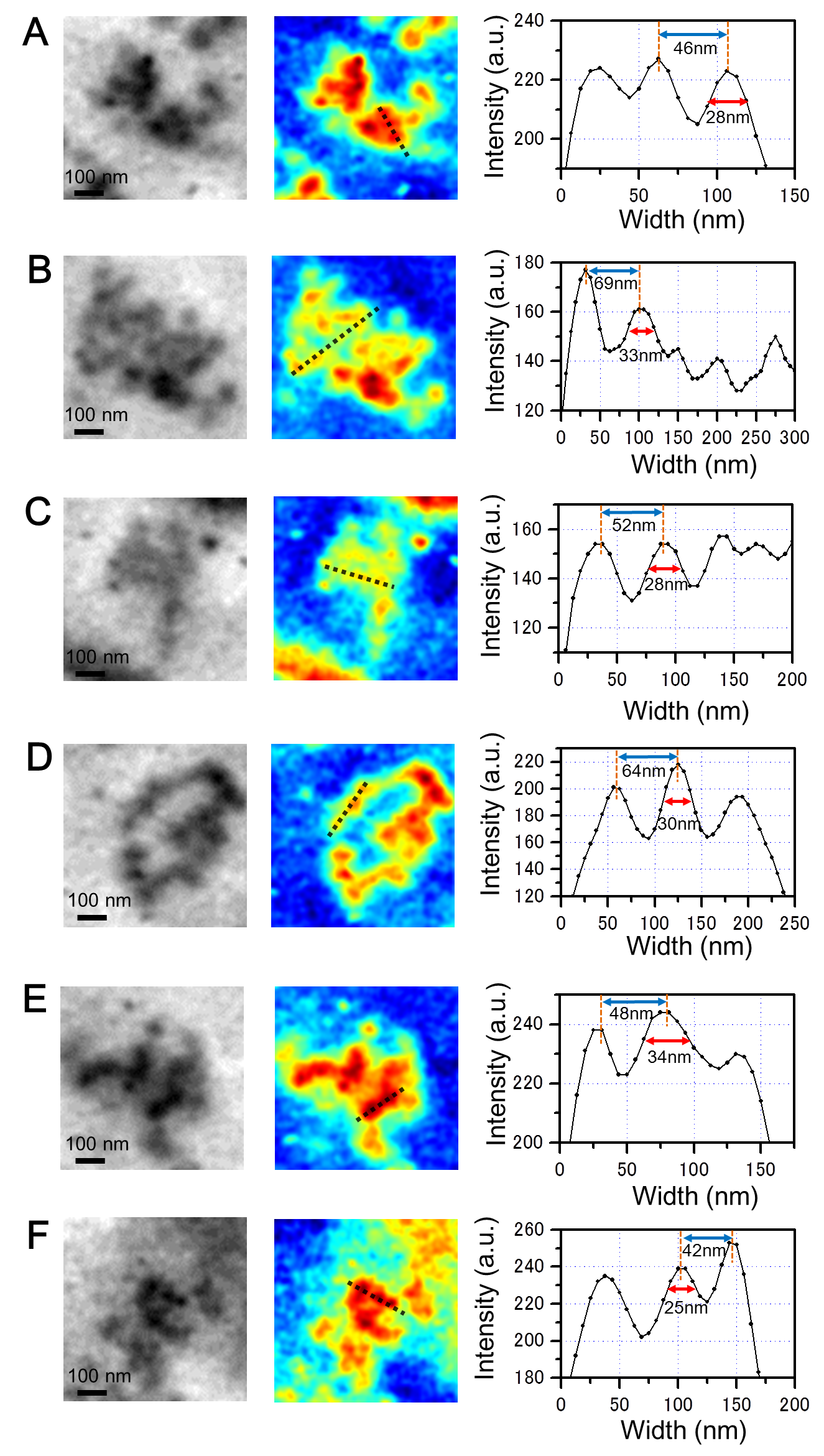

Supplement: S7 Fig — (A–F) Enlargements of six adhesion cores containing small granules observed by the SE-ADM system (15,000× magnification, 6-kV EB acceleration, 7 mm working distance, −9 V bias). The left and central panels show the enlarged images and their intensity-inverted pseudo-color maps, respectively. The right panels are the line plots of the integrin granular regions along the dotted lines in the corresponding pseudo-color maps. The diameter and separation of the adhesion particles (30.4 ± 4.0 and 53.9 ± 11.1 nm, respectively) were averaged over eight adhesion cores selected from these images and those in Fig 4. All scale bars are 100 nm. (TIF) [file pone.0204133.s007.tif]

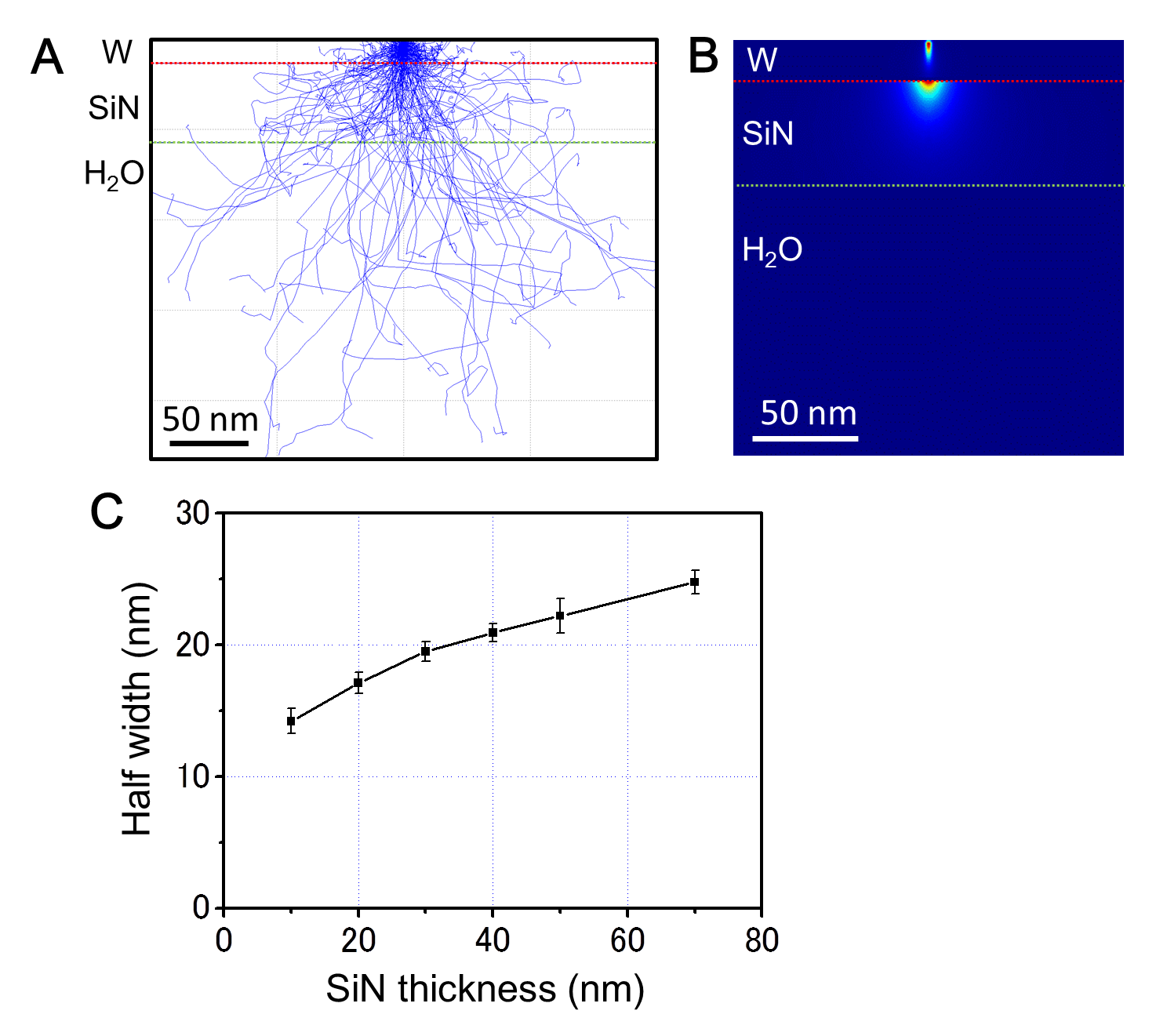

Supplement: S8 Fig — (A) Monte Carlo simulation of the W-coated SiN film executed in CASINO ver. 2.42 [35], showing the electron trajectory area of the 50-nm-thick SiN film on the 15-nm-thick W layer. The EB spot diameter was 3 nm. At a beam-accelerating voltage of 4 kV, almost all of the electrons passing through the W layer were absorbed by the SiN film. (B) Cross-section of the energy deposited in the W-coated SiN film by the 4 kV electrons in the EB, normalized by each layer of the film. (C) Full-width-at-half-maximum of the energy absorbed in the SiN film as a function of film thickness (10–70 nm). The acceleration voltage and W layer thickness were fixed at 4 kV and 15 nm, respectively. Plotted are the averages and standard deviations of five simulations at each setting. The spatial resolution of the SE-ADM system depends on the width of the absorbed energy in the SiN film. (TIF) [file pone.0204133.s008.tif]

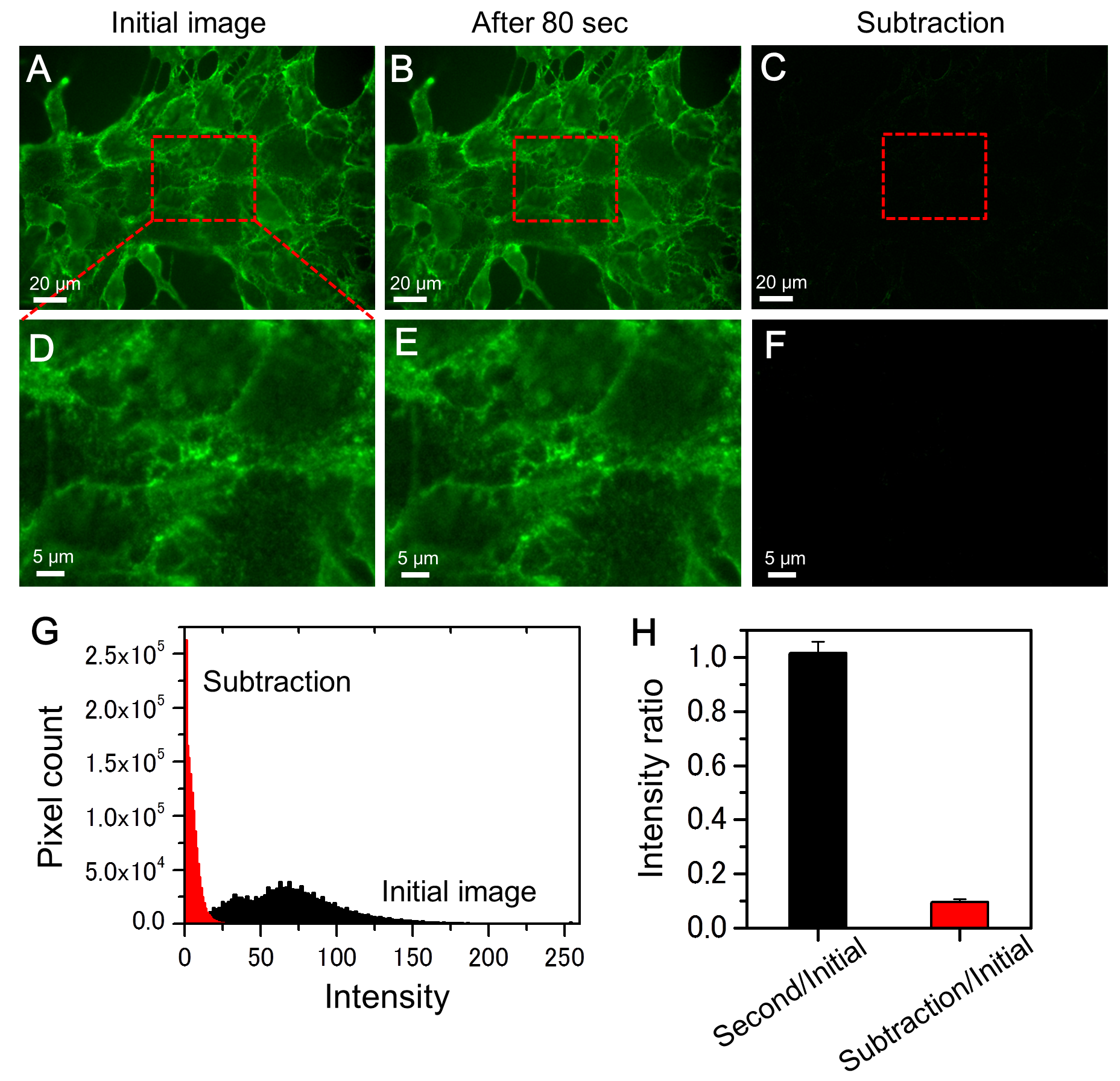

Supplement: S9 Fig — (A) Initial fluorescence image of 4T1E/M3 cells stained with biotin-conjugated rabbit anti-integrin β1 antibody and FITC-conjugated streptavidin and observed by an optical fluorescence microscope at 400× magnification with a green fluorescence filter. (B) Fluorescence image of the same region after 80 s. (C) Intensity image of the initial minus the later image. The integrin structure has almost completely disappeared. (D), (E), and (F) Enlarged images of the integrin β1 spots enclosed in the red squares in (A), (B), and (C), respectively, implying that the adhesion cores are almost immobilized under our experimental conditions. (G) Image-intensity distributions of the initial (black) and subtraction (red) images. The intensity of the subtraction image is below 25 at almost all locations. (H) Ratios of summation intensities between the initial and later images (black bar) and between the initial and subtraction images (red bar). The mean ratios, determined from 10 images, are 1.014 ± 0.0425 and 0.096 ± 0.011, respectively. The fluorescence intensities of the first and second images are almost identical (the subtraction image is 1/10 as intense as the initial image). Scale bars: 20 μm in (A–C) and 5 μm in (D–F). (TIF) [file pone.0204133.s009.tif]
